# Human equivalent doses of l-DOPA rescues retinal morphology and visual function in a murine model of albinism

**DOI:** 10.1038/s41598-023-44373-3

**Published:** 2023-10-11

**Authors:** Aida Sanchez-Bretano, Eloise Keeling, Jennifer A. Scott, Savannah A. Lynn, Sudha Priya Soundara-Pandi, Sarah L. Macdonald, Tutte Newall, Helen Griffiths, Andrew J. Lotery, J. Arjuna Ratnayaka, Jay E. Self, Helena Lee

**Affiliations:** 1 Clinical and Experimental Sciences, Faculty of Medicine, University of Southampton, Sir Henry Wellcome Laboratories, Southampton University Hospital, South Block Mail Point 806, Level D, Southampton, SO16 6YD UK; 2 Eye Unit, University Hospital Southampton NHS Foundation Trust, Tremona Road, Southampton, SO16 6YD UK

**Keywords:** Drug discovery, Neuroscience, Diseases

## Abstract

l-DOPA is deficient in the developing albino eye, resulting in abnormalities of retinal development and visual impairment. Ongoing retinal development after birth has also been demonstrated in the developing albino eye offering a potential therapeutic window in humans. To study whether human equivalent doses of l-DOPA/Carbidopa administered during the crucial postnatal period of neuroplasticity can rescue visual function, OCA C57BL/6 J-c2J OCA1 mice were treated with a 28-day course of oral l-DOPA/Carbidopa at 3 different doses from 15 to 43 days postnatal age (PNA) and for 3 different lengths of treatment, to identify optimum dosage and treatment length. Visual electrophysiology, acuity, and retinal morphology were measured at 4, 5, 6, 12 and 16 weeks PNA and compared to untreated C57BL/6 J (WT) and OCA1 mice. Quantification of PEDF, βIII-tubulin and syntaxin-3 expression was also performed. Our data showed impaired retinal morphology, decreased retinal function and lower visual acuity in untreated OCA1 mice compared to WT mice. These changes were diminished or eliminated when treated with higher doses of l-DOPA/Carbidopa. Our results demonstrate that oral l-DOPA/Carbidopa supplementation at human equivalent doses during the postnatal critical period of retinal neuroplasticity can rescue visual retinal morphology and retinal function, via PEDF upregulation and modulation of retinal synaptogenesis, providing a further step towards developing an effective treatment for albinism patients.

## Introduction

Oculocutaneous albinism (OCA) affects approximately 1:17,000 people globally^1^, with a lower rate in the United States (1:37,000)^2^ and the highest rate found in Panamanian and Colombian indigenous people (6.3:1000)^3,4^. It is a genetic disorder characterized by the partial or complete absence of melanin biosynthesis^5–8^. Consequently people with OCA often have fair skin and hair, an increased susceptibility to the effects of ultraviolet radiation on their skin^3^, abnormalities of retinal development, nystagmus, strabismus, refractive errors, optic nerve misrouting and visual impairment^9–13^. The severity of the condition can vary and some people have all of these features, and others only some. However, the vast majority of people with OCA are have significant visual impairment and are registered as sight impaired (Certificate of Visual Impairment, CVI) due to the severity of the ocular manifestations^14^. To date, there is no effective treatment.

OCA is caused by numerous genes and often has a complex genotype. OCA1 is the most frequent subtype of OCA, accounting for almost 50% of cases worldwide^3,15,16^. It occurs as a consequence of mutations in the *TYR* gene^6,15,17^, that encodes the enzyme responsible for transforming tyrosine to l-DOPA, and l-DOPA to melanin in pigmented cells including melanocytes^18–20^ and the retinal pigment epithelium (RPE)^21–23^. In mice, there is a physiological peak of l-DOPA in the RPE from the early stages of retinal development through to maturation in the late postnatal phase (at ~ 4 weeks of age)^24^. The lack of l-DOPA in the RPE in OCA1 during this critical period is thought to result in abnormal retinal development and poor visual function^24^.

Retinal development is considered an overlapping process of two different phases: *neurogenesis*, which includes proliferation and differentiation of cells into the different retinal cellular types^25^, and *synaptogenesis* which establishes the retinal circuitry network. These two processes continue after birth in a postnatal developmental window. The study from Young^25^ described the timing in which mitosis takes place in the murine developing retina, with evidence of overlap between the division and differentiation of some retinal cell populations. According to this study, the end of the proliferation phase takes place around postnatal day (PND) 12 in mice^25^, although the differentiation phase continues for longer^26^. The synaptogenesis phase starts after eye opening (at PND 12) and continues for several weeks, coinciding with the physiological peak of l-DOPA in the RPE^24,27^.

Our previous study in a murine model of OCA1, indicated that 28 days of l-DOPA supplementation (10 mg/kg) during the postnatal critical period of retinal development caused an improvement in retinal morphology and function during and after the end of the treatment^14^. This suggested that mimicking physiological levels of l-DOPA during development could improve vision in children with albinism. However, this dose (HED of 4.35 mg/kg) is more than the maximum l-DOPA dose recommended by the UK British National Formulary for Children. Therefore, in this study, we selected human equivalent doses (HED) that have been established for use in infants and young children diagnosed with infantile dystonia and amblyopia^28–33^ to determine its potential value for treating OCA1 patients. To do so, we studied the optimal conditions for l-DOPA treatment that can maximally rescue visual function in a murine model of OCA1, in preparation for future clinical trials.

## Materials and methods

### Animals

The C57BL/6 J-c2J (*Tyr*^*c-2j*^) null OCA1 mice were chosen for this study as it is an established model for testing potential treatments for OCA1^14,24^. To analyse the effect of l-DOPA on retinal development in OCA1 mice, pigmented wild type (WT) mice, with the same genetic background and lacking the *TYR*^*c-2j*^ gene mutation (C57BL/6 J; C57) were used as a control. Mice were housed at the Biomedical Research Facility (BRF) at the University of Southampton, UK. All mice were maintained in conventional cages under controlled light conditions (12:12) hours of light/dark cycle with food and water available ad libitum. Weight was monitored at all the experimental time points. All studies were performed in accordance with the ARVO Statement for the use of animals in ophthalmic and vision research, in accordance with the NC3Rs ARRIVE guidelines, and after approval was obtained from Animal Welfare and Ethical Review Body, University of Southampton (project licence number: P474F5E4D) under the UK Animals (Scientific Procedures) Act (1986). All possible steps were taken to avoid animal suffering at each stage of the experiment. For termination procedures, mice were sacrificed with 0.1 ml pentobarbital and transcardially perfused with 0.9% NaCl (Sigma Aldrich, UK) containing 5 units/ml heparin sulphate (CP Pharmaceuticals, UK) prior to enucleation. Experimenters were blinded to the groups from allocation through to data analysis. N-numbers for each group are shown in Table 2.

### l-DOPA treatment

WT and OCA1 mice were supplemented with a solution containing l-DOPA (Sigma Aldrich, UK) and carbidopa (Sigma Aldrich, UK), dissolved in ddH2O drinking water with ascorbate (2.5 mg/ml) to prevent oxidation, for 28 days starting on PND15 (Table 1). l-DOPA is co-administered with carbidopa in order to minimize systemic side effects that can be experienced from l-DOPA exposure alone^34^. The doses selected were based on current l-DOPA use in amblyopia^28^ and were calculated as HED following the protocol described by Nair and Jacob^35^. The optimum length for the treatment was subsequently assessed by treating OCA1 mice with the selected dose from PND 15 for 2, 4 or 8 weeks. N-numbers (whole animals) for each treatment group are shown in Table 2.

**Table 1:** l-DOPA dosages used in the experiment.

	l-DOPA*	Carbidopa*	l-DOPA HED
Low dose	6.15 mg/kg	1.5375 mg/kg	0.50 mg/kg TDS
Medium dose	9.348 mg/kg	2.337 mg/kg	0.76 mg/kg TDS
High dose	12.3 mg/kg	3.075 mg/kg	1.00 mg/kg TDS

*HED* human equivalent dosage.

*Assuming an average daily water consumption of 4 ml in an adult 25 g mouse.

**Table 2:** N-numbers for each experimental group.

		CALB (M)	CALB (F)	C57 (M)	C57 (F)
Exp 1	Low	9	9	8	10
	Medium	13	9	26	25
	High	5	18	8	19
	Control	13	32	15	10
Exp 2	Short	16	12		
	Medium	23	15		
	Long	13	9		
	Control	27	11		

### Dose-optimisation study

**Figure 1:**
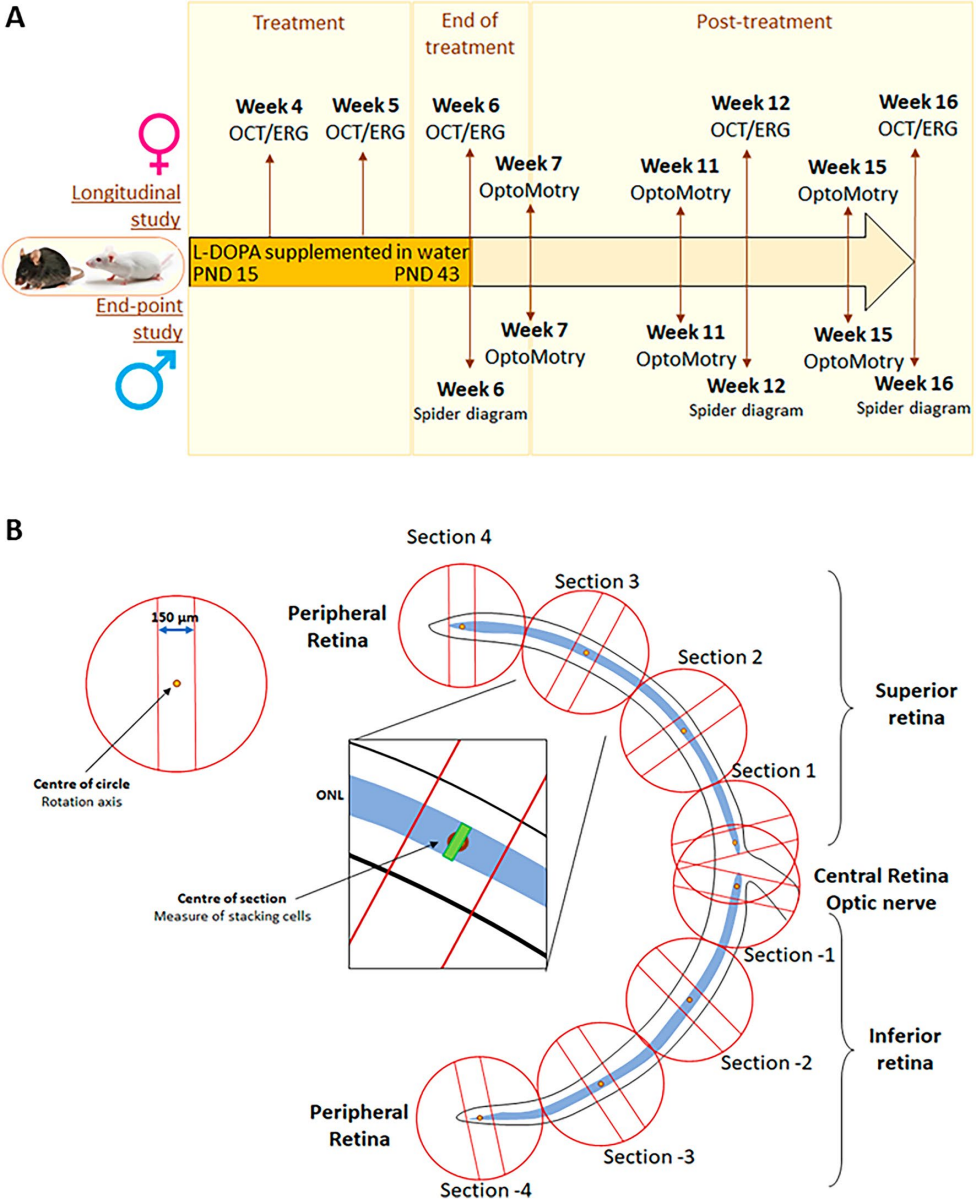
Experimental design and histology measurements. (**A**) Experimental design. Male and female WT and OCA1 mice were treated with normal water or water supplemented with l-DOPA for 28 days from PND 15. A longitudinal study was performed with females with retinal morphology and function assessments at weeks 4, 5, 6, 12 and 16 and spatial frequency thresholds measured at weeks 7, 11 and 15. All females were sacrificed at week 16 to obtain eye samples for post-mortem studies. An end-point experiment was performed in parallel with male mice where retinal morphology and function were assessed at only one time point at week 6, 12 or 16. Each male was sacrificed after its retinal assessments to obtain eye samples for post-mortem studies. Therefore, in males, spatial frequency thresholds were analysed up until the point of sacrifice of the different mice. Specifically, males with retinal assessments at week 6 did not contribute to spatial frequency studies, whilst those with retinal assessments at week 12 had their spatial frequency thresholds measured at weeks 7 and 11 and those with their retinal assessments at week 16 underwent OptoMotry at weeks 7, 11 and 15. (**B**) Diagram describing the approach used to count cell number and cell stacking. Each retina was divided into 8 sections with a circular template that included a space of 150 µm wide. A dot at the centre of the circle acted as the rotational axis. Four circles were located in both the superior and inferior retinas starting with the closest to the optic nerve, located touching the end of the ONL at the optic nerve level and the furthest to the optic nerve touching the end of the ONL at the periphery of the retina. From there, the other two circles were in contact, equidistant to the others, dividing each superior and inferior retina into four sections, each named from 1 to 4 according to its distance to the optic nerve (from closer to further) and using a negative symbol to differentiate inferior retina from superior. The dot in the middle of the circle was positioned in the centre of the ONL and the circles were rotated until the internal straight lines were perpendicular to the limits of the ONL. Cells were counted in the spaces within the internal straight lines of each section and the stacking of cells was counting in the centre of each section, indicated as a green rectangle in the image. *WT* wild type, *OCA1* oculocutaneous albinism type 1, *ONL* outer nuclear layer.

l-DOPA treatment was administered to WT and OCA1 mice. Female mice were used for ongoing longitudinal studies and had their retinal morphology evaluated by Optical Coherence Tomography (OCT) and histology and retinal function measured by Electroretinogram (ERG) at 4, 5, 6, 12 and 16 weeks PNA. Visual acuity was also taken by OptoMotry at 7, 11 and 15 weeks PNA. Male mice, which were unable to tolerate multiple anaesthetic doses were used for endpoint studies and their retinal morphology and function was measured at a single time point prior to culling, at either 6, 12 or 16 weeks PNA. Visual acuity was also measured at 7 and 11 weeks for the 12 week cohort, and at 7, 11 and 15 weeks for the 16 week PNA group. All female mice were culled at 16 weeks PNA and these, along with the male eyes from 6, 12 or 16 weeks PNA were collected and prepared for post-mortem studies as outlined below. The experimental design is detailed in Fig. 1 with N-number for each treatment group are shown in Table 2.

### Length of treatment study

l-DOPA was administered to both male and female OCA1 mice and WT mice with the selected optimum dose from PND15 for 2 weeks, 4 weeks or 8 weeks. Visual acuity was assessed at the end of treatment, at 6 weeks after treatment and 16 weeks PNA. At 16 weeks, eyes were collected and prepared for post-mortem studies at outlined below. The experimental design is shown in Fig. 1 and N-numbers for each treatment group are shown in Table 2.

### Optical coherence tomography (OCT)

Mice were anesthetized with an intraperitoneal (IP) injection of 1 mg ketamine (Bayer PLC, UK and 0.005 mg dexmedetomine hydrochloride (Centaur Services, UK) per 10 g body weight and were placed in the dark until motor function ceased. Pupils were dilated using tropicamide 1% w/v eye drops (Chauvin Pharmaceuticals Ltd, UK) and phenylephrine hydrochloride 2.5% w/v eye drops (Chauvin Pharmaceuticals Ltd, UK) and corneas lubricated with a coupling gel (Clinitas gel; Alcon, UK). Artificial tears (Viscotears; Alcon, UK) were used throughout the procedure to maintain corneal moisture and clarity. Animals were wrapped in surgical gauze and positioned comfortably within a stereotactic rotational cassette, with their heads aligned on a bite bar, in an animal imaging mount. The eye was positioned in line with the mouse retina lens using the multiaxial rodent alignment stage apparatus. The Leica Envisu R2200 VHR SDOIS Mouse Imaging System (BioptigenInc, USA) was used to obtain 1.4 mm volumetric scans (consisting of 100 B-scans and 1000 A-scans per B-scan) through a 50 degrees field of view, centred on the optic disc.

Automatic segmentation of the individual retinal layers was carried out using InVivoVue 3.3 Diver software (Leica Microsystems, UK). This software performs a full volume, 8-layer segmentation (retinal nerve fibre layer (RNFL), combined ganglion cell layer + inner plexiform layer (IPL), inner nuclear layer (INL), outer plexiform layer (OPL), outer nuclear layer (ONL), photoreceptor inner segment (IS), photoreceptor outer segment (OS) and retinal pigment epithelium (RPE)) of the retina and has previously been shown to be accurate^36,37^.

### Electroretinogram recordings (ERG)

Scotopic full-field electroretinogram (ERG) recordings were obtained using the Phoenix Micron III Retinal Imaging System and second-generation Image-Guided ERG system designed for rodents (Phoenix Research Labs, USA). Mice were dark adapted for 12 h overnight and subsequently handled under dim red-light illumination in red Leddy IVC cages (Techniplast, UK) in a controlled IVC air flow system. Mice were anesthetised with 1 mg Ketamine (Bayer PLC, UK) and 0.005 mg dexmedetomidine hydrochloride (Centaur Services, UK) per 10 g body weight. Upon cessation of motor function, pupils were dilated as described above. During anaesthesia, ocular hydration was maintained via periodic application of Viscotears (Alcon, UK) Animals were positioned on a heated pad to maintain constant body temperature and connected to a subdermal ground electrode (in the lumbar region), a reference electrode (on the head) and an active corneal contact electrode placed by positioning the cornea onto the gold-plated objective lens. Scotopic full-field ERGs were recorded by stimulating with a 1.5 mm (approximately 7.7-disc diameters) circular LED white light stimulus diameter, at a flash strength of 6.8log cd-s/m^2^ on to the central retina white LED light for 1 ms. Two sweeps per eye were carried out with an interval time of 120 s. ERGs were visualised in the V3 Phoenix LabScribe ERG software suits (Phoenix Research Labs, Pleasanton, CA, USA). A-wave and B-wave amplitudes were calculated as the measurement from baseline to the A-wave trough and the A-wave trough to the B-wave peak respectively. The implicit time (the time interval between stimuli onset and the wave peak) for both the A and B waves were also recorded. All measurements were conducted inside a 6-panel aluminium copper mesh Faraday cage (Micro Control Instruments Ltd., Framfield, UK) to prevent external interference.

### OptoMotry

Spatial frequency thresholds were assessed for both female and male mice at 7, 11 and 15 weeks PNA by recording opto-kinetic tracking (OKT) using the OptoMotry system (Cerebral Mechanics Inc, Lethbride, Alberta, Canada). Mice had initial training sessions to familiarise them with the machine and were acclimatised for 5 min prior to experimentation. During the test procedure, awake and freely moving mice were placed on a platform located in the centre of a chamber surrounded by four connected liquid crystal display (LCD) monitors with a motioned black and white sine-wave grating drawn in 3D space rotating around the animal at a speed of 12 deg/sec. A camera was positioned overhead to allow for monitoring of visually evoked reflexive head and neck movements. The spatial frequency of the grating was systematically increased (0.03c/d) at 100% contrast in the OptoMotry HD software to determine the spatial frequency at which the subject was no longer able to track. If a stimulus was detectable mice initiated a head tracking as defined by a smooth reflexive head movement in the direction of rotation (clockwise, counter clockwise or up and down) with comparable velocity to the speed of the sine-wave gratings. The assessments took place during the second half of the light phase and each assessment lasted around 10 to 15 min per mouse.

### Quantitative morphometric analysis

To measure the number of nuclear layers in the ONL, eyes were collected from male mice at 6, 12 and 16 weeks PNA, and from female mice at 16 weeks PNA. Samples were rapidly immersed in a methanol/glacial acetic acid (97:3 v/v) solution in a dry ice bath and kept at − 80 °C for a minimum of 4 days to allow the methanol to substitute the total content of water in the samples. The eyes were then embedded in paraffin and cut into 5 μm sections. Sections containing the optic nerve were selected and stained with haematoxylin/eosin (H&E). Briefly, sections were soaked in Mayer’s Haematoxylin for 10 min and rinsed in water for 5 min. 0.03% acid alcohol was applied for a further 10 min before being rinse in tap water for a further 5 min. Slides were then stained in 0.5% Eosin for 5 min and rinsed briefly in ddH_2_O before being submerged in an ethanol dehydration gradient (50%, 70%, 90%, and 100% ethanol twice). Xylene was then added to the slides for 5 min before they were mounted on coverslips with DPX mountant (Sigma Aldrich, UK). Slides were imaged using an Olympus VS110 virtual slide scanning microscope at × 20 magnification (Olympus, UK). Each retinal hemisphere was divided into 8 equal segments, 4 on the superior and 4 on the inferior side of the optic nerve (Fig. 1B). The number of layers of nuclei and the total number of nuclei in the ONL were counted at four equidistant locations (sections of 150 µm wide) in both superior and inferior parts of the retina, averaged, and quantified using FIJI software, as illustrated by the diagram in Fig. 1B. The area of the nuclei in the ONL layer was measured using FIJI software. Briefly, images were opened in FIJI, and the scale bar incorporated based on the imaging parameters. The images were converted into a 32-bit format and the threshold adjusted by using the Shanbhag filter. A watershed filter was used to clearly define the separation between cell nuclei. The ONL area was then selected and the particle size for 50 randomly selected cells in each retina was determined.

### Immunofluorescence

Eye sections were embedded in paraffin and frozen as described previously. 50 µm sections were cut and collected on slides. Slides were dewaxed by heating in a 60 °C oven for 15 min. Samples were rehydrated through graded alcohol washes of Xylene 1, Xylene 2, 100% Industrial Methylated Spirit (IMS) 1, 100% IMS 2, 95% IMS, 70% IMS, and 50% IMS. Slides were then washed twice in PBS and blocked for 1 h in a solution containing 0.1% Triton X-100 and 5% BSA in PBS. Primary antibodies against rhodopsin (1:100 Ab3267, Abcam, UK) and class- III β-tubulin (1:200 Ab18207, Abcam) were incubated overnight at 4 °C. Then, slides were washed twice in PBS, incubated with secondary antibodies (1:200, Alexa Fluor 488 and Alexa Fluor 568, Life Technologies) for 1 h at room temperature in the dark and washed in PBS to remove unbound antibodies before mounting with Vectashield DAPI (1:500). A separate set of negative control slides were only treated with the secondary antibody. Samples were imaged using a Leica DM4B (Leica Microsytems, UK) at × 63 magnification, saved as TIFF files and observed through FIJI software (FIJI, UK).

### Western blot

Eyes from 16 weeks old female mice and from 6, 12 and 16 weeks old male mice were obtained and immediately frozen in dry ice. Protein levels from these samples were quantified by western blot. The lens was removed from the eyes before they were homogenized in 100 µl of 1 × RIPA buffer (0.5 M Tris–HCl, pH 7.4, 1.5 M NaCl, 2.5% deoxycholic acid, 10% NP-40, 10 mM EDTA; Sigma, UK) prepared with 1 × proteinase (Sigma, UK) and 1 × phosphatase inhibitor cocktail (Sigma, UK) using a syringe. Samples were maintained on ice and vortexed every 10 min for 1 h. Homogenates were subsequently centrifuged at 14,000 rpm for 20 min at 4 °C and the supernatant was collected and stored on ice. The total concentration of protein in the homogenates was quantified by a BCA Assay (Pierce, UK) according to the manufacturer’s instructions. For the western blot assay, 50 µg of protein were prepared in 2 × Novex Tris–Glycine SDS Sample Buffer (ThermoFisher, UK) and loaded into a 12% Mini-PROTEAN® TGX™ precast protein gel (Bio-Rad). Electrophoresis ran for 10 min at 90 V initially , and then for another 40 min at 150 V. 1 × premixed electrophoresis buffer: 25 mM Tris, 192 mM glycine, 0.1% SDS, pH 8.3 (#1610732, Bio-Rad) was used as the running buffer. Spectra Broad Range Protein Ladder (Fisher, UK) and Chameleon® Duo Pre-stained Protein Ladder (Li-Cor, UK) were used as molecular markers. Following electrophoresis, proteins were transferred to a 0.2 µm pore-size nitrocellulose membranes (Bio-Rad, USA) using the Trans-Blot® Turbo™ transfer system (Bio-Rad, USA), in 1 × premixed Transfer buffer (20% ethanol, 60% nanopure water; Bio-Rad, USA) and membranes were blocked in Intercept® (TBS) Blocking Buffer (Li-Cor, UK). Detection of the target proteins was performed by overnight incubation of membranes in Intercept® (TBS) Blocking Buffer (Li-Cor, UK) containing primary antibodies PEDF (1:500; R&D Systems), B3-tubulin (1:1000; Abcam), or Syntaxin-3 (1:100; Abcam). Vinculin (Cell Signaling) was used for normalization, as previously used successfully in murine tissues^38^. Membranes were then washed in TBS containing 0.1% tween 20 and incubated with secondary antibodies IRDye® 680 and 800 (Li-Cor, UK) depending on the primary antibody being detected. Finally, membranes were imaged by the Odyssey FC system (Li-Cor, UK) and protein bands were quantified by densitometry using Image Studio software (Li-Cor, UK). The target protein content of each band was normalized to the corresponding vinculin content and protein levels in the different experimental groups were calculated as a fold-change with respect to the control group (C57, non-treated mice, normalized protein expression).

### Statistics

Data obtained from the OCT, ERG, OptoMotry and histology assessments were assessed for normality of variance and homoscedasticity. The different variables studied (retinal layers thickness obtained by OCT; cell count, stacking and area obtained by histological analyses; spatial frequency thresholds measured by OptoMotry; and a- and b-wave amplitudes and implicit times measured by ERG) were analysed using a general linear model in SPSS with “genotype”, “age” and “treatment” as fixed factors, followed by a Bonferroni post-hoc test. Results of statistical tests are provided in Supplementary Table 1. The n numbers for each of the variables and groups, as well as values for mean and standard deviations (SD) are included in the Supplementary Tables 2–7. When the data was not normally distributed, a two-step transformation of the data was performed on SPSS prior to analysis^37^. Sex differences were analysed using *t* tests. To determine if sustained, significant improvements in retinal function can be achieved with different lengths of treatment, an independent samples Kruskal–Wallis test with Bonferroni post hoc correction was carried out. Western blot densitometry results were analysed using two-way ANOVA. Differences of P < 0.05 were considered statistically significant.

### Ethics approval

All experiments were performed in accordance with the ARVO Statement for the use of animals in ophthalmic and vision research, in accordance with the NC3Rs ARRIVE guidelines, and after approval was obtained from Animal Welfare and Ethical Review Body, University of Southampton (project licence number: P474F5E4D) under the UK Animals (Scientific Procedures) Act (1986). All possible steps were taken to avoid animal suffering at each stage of the experiment.

## Results

### Human equivalent doses of l-DOPA have no effect on body weight and were not sex dependent

The weight of the mice was measured at all experimental time points and no differences were observed between WT and OCA1 mice in any of the study conditions (treated and untreated groups, Supplementary Fig. 1). Both phenotypes increased their body weight normally throughout the experiment (Supplementary Fig. 1).

Longitudinal results obtained from females were compared with those obtained from the endpoint studies of male mice to investigate possible effects in sex differences. ERG, OCT and OptoMotry results revealed no differences between sexes due to treatment (Supplementary Figs. 2, 3, 4 and 5).

### l-DOPA has no effect on WT murine retinal morphology or visual acuity

**Figure 2:**
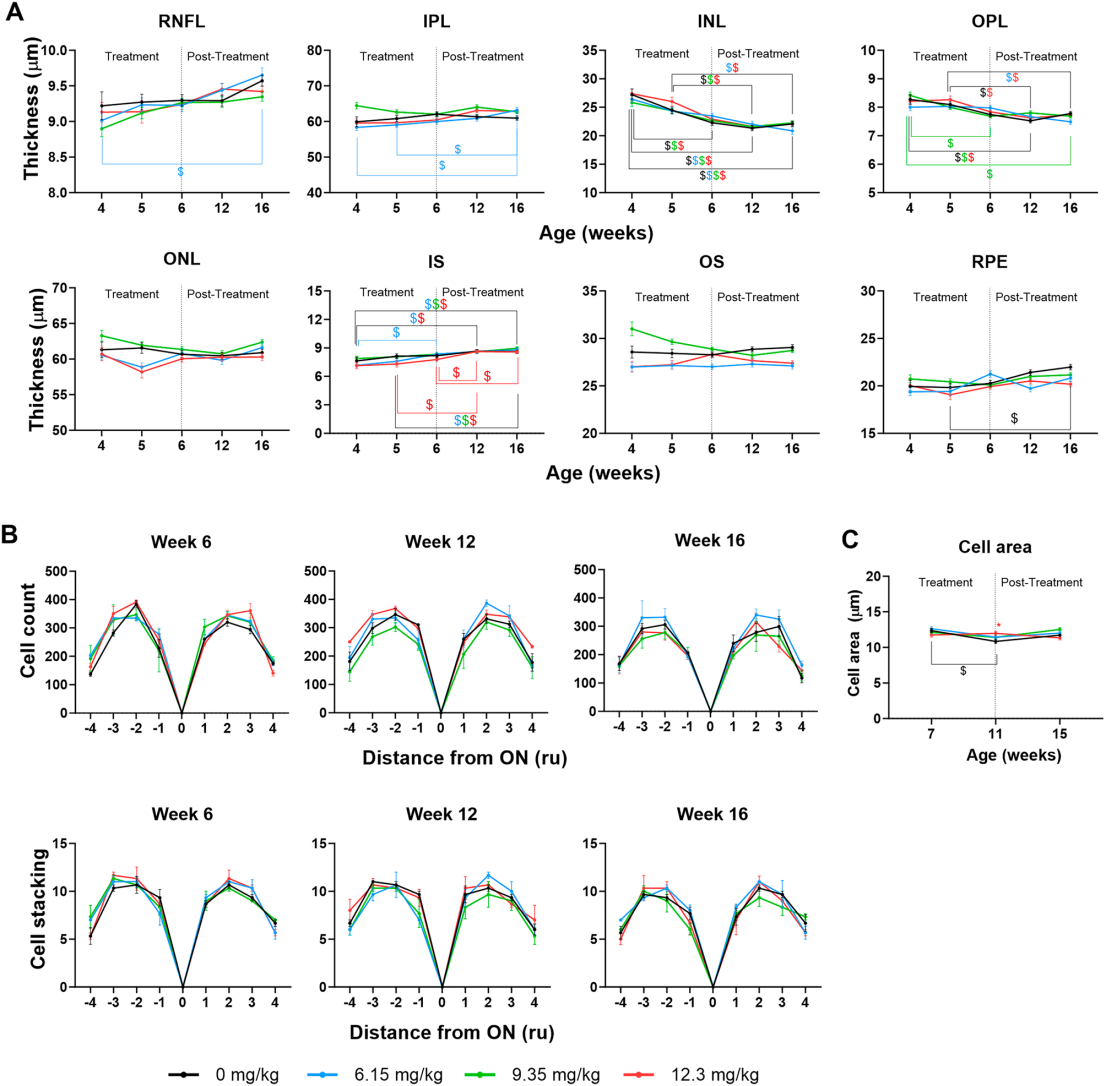
l-DOPA effects on WT mice retinal morphology. (**A**) Thickness of the different layers of the retina of WT treated with different doses of l-DOPA along the experiment measured by OCT. (**B**) ONL cell count (upper panels) and cell stacking (lower panels) on WT mice. (**C**) Cell area of WT mice. Results are shown as mean ± SEM. Asterisks indicate significant differences (P < 0.05) between non-treated and l-DOPA-treated WT mice. The colour of the asterisk indicates the treated group (i.e. blue asterisk corresponds with WT mice treated with l-DOPA at 6.15 mg/kg) that is statistically different from non-treated WT mice. The dollar symbols indicate significant differences (P < 0.05) across time in the same group of mice indicated by the colour of the symbol.

**Figure 3:**
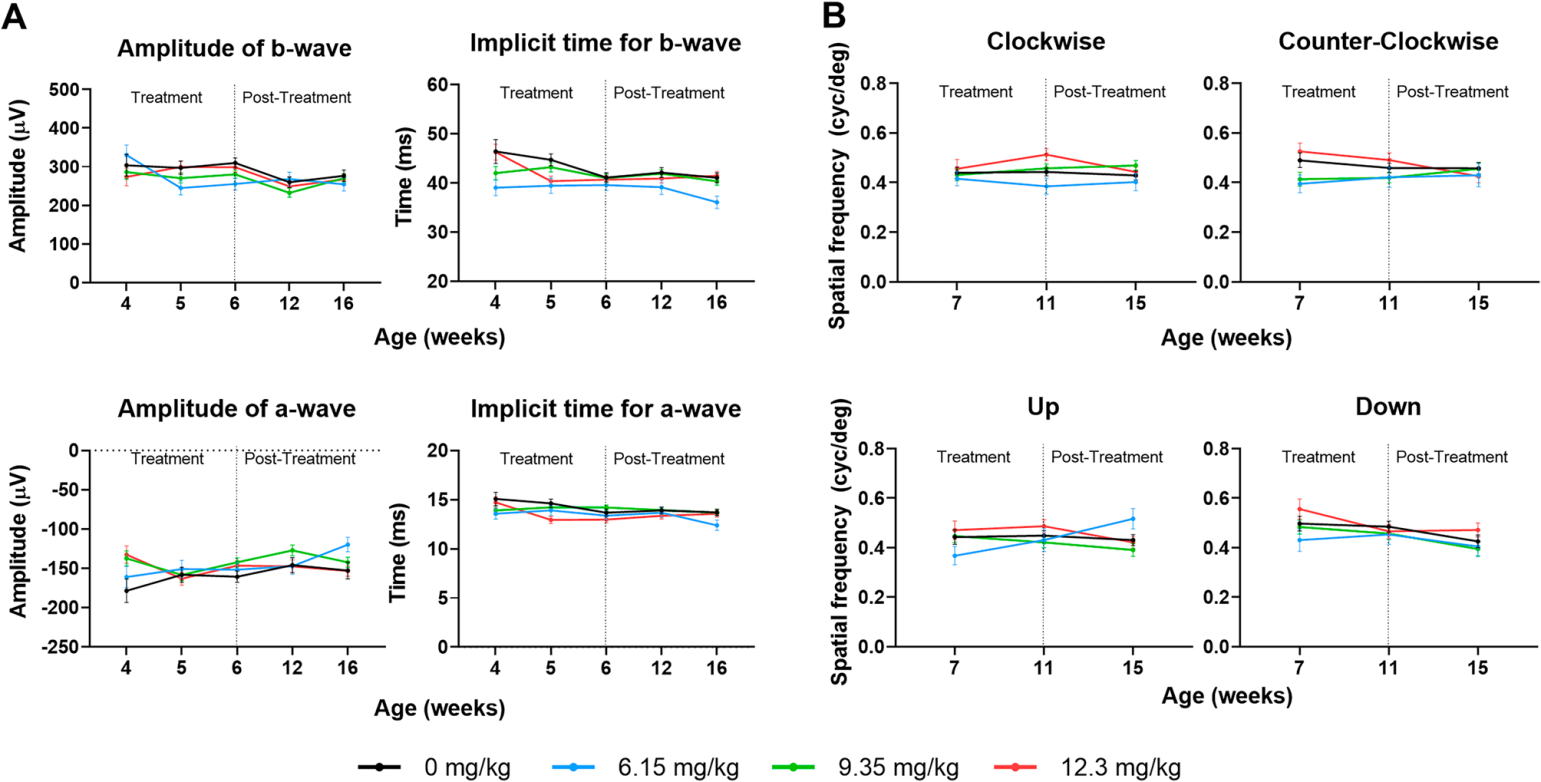
l-DOPA effects on WT mice visual function. (**A**) Retinal response to a light stimulus in WT mice throughout the experiment including a- and b-wave amplitudes (left panels) and a- and b- wave implicit times (right panels) as measured by ERGs. (**B**) Spatial frequency thresholds for WT mice treated with l-DOPA throughout the experiment as measured by OptoMotry. Results are shown as mean ± SEM. No significant differences were found.

WT mice treated with l-DOPA at the three different doses showed no significant differences in retinal morphology (Fig. 2, Supplementary Tables 1, 2, 3 and 4), function (Fig. 3A, 7A, Supplementary Table 5) or spatial frequency thresholds (Fig. 3B, Supplementary Table 6). The only significant difference identified was in WT mice treated with 6.15 or 12.3 mg/kg of l-DOPA, showing a significantly thinner IS layer measured by OCT during the first two weeks following treatment (4 and 5 weeks PNA) which returned to normal thickness values at 6 weeks PNA.

### Age affects retinal morphology but not visual function in WT mice

RNFL thickness tended to increase throughout the time course of the experiment, but only reached statistical significance in WT mice treated with the lowest dose of l-DOPA (6.15 mg/kg) between 4 and 16 weeks PNA (Fig. 2A, Supplementary Table 2).

A significant increase in thickness was observed in WT mice treated with l-DOPA 6.15 mg/kg at the level of the inner plexiform layer (IPL), in all l-DOPA-treated WT mice at the level of the IS, and in untreated WT mice at the level of the RPE (Fig. 2A, Supplementary Table 2).

The thickness of the inner nuclear layer (INL) and the outer plexiform layer (OPL) decreased significantly throughout the experiment in both treated and untreated WT mice (Fig. 2A, Supplementary Table 2). There were no significant time-related changes in ONL, OS and RPE thickness measurements.

Histological analyses showed that from week 4 to 16 weeks PNA, there was no significant change in cellular morphology in WT mice (Fig. 2B and C, Supplementary Tables 2, 3 and 4), with only a small decrease in cell area at 11 weeks PNA in untreated WT mice, which returned to normal levels by 16 weeks PNA (Fig. 2C).

There were also no significant differences throughout the study in the electrical responses of the retina (Fig. 3A and Supplementary Table 5) and in spatial frequency thresholds recorded from WT mice (Fig. 3B and Supplementary Table 6).

### l-DOPA partially corrects the differences in retinal morphology seen in OCA1 mice compared with WT

**Figure 4:**
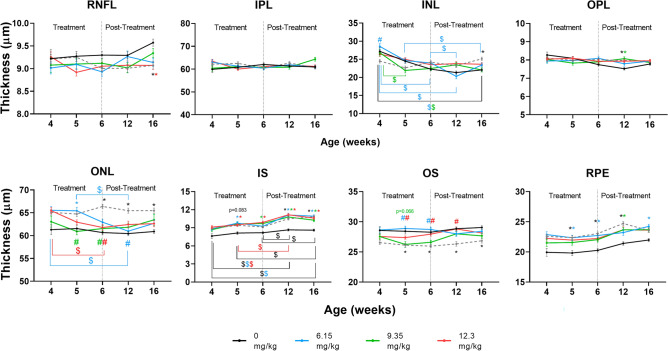
l-DOPA effects on retinal layers thickness in OCA1 mice. Untreated OCA1 mice showed an increase in thickness of the outer nuclear layer (ONL), inner segment of the photoreceptors (IS) and retinal-pigmented epithelium (RPE) and a decrease in thickness of the outer segment of the photoreceptors (OS). All different l-DOPA doses tested corrected the thickness of ONL and OS in OCA1 mice. There was a slight improvement in the RPE thickness in mice treated with any dose of l-DOPA tested and there was no significant effect of l-DOPA treatments in IS thickness. Results are shown as mean ± SEM. Asterisks indicate significant differences (P < 0.05) between non-treated WT mice and the different OCA1 mice groups. The colour of the asterisks indicates the albino group (i.e. blue asterisk corresponds with OCA1 mice treated with l-DOPA at 6.15 mg/kg) that is statistically different from non-treated WT mice. Hashtags indicate significant differences (P < 0.05) between treated OCA1 mice when compared with untreated OCA1 mice. The colour of the hashtag indicates the albino group that is statistically different from untreated OCA1 mice. The dollar symbols indicate significant differences (P < 0.05) across time in the same group of mice indicated by the colour of the symbol.

OCA1 mice exhibited significant differences in retinal layer thickness measurements compared with untreated WT mice. OCA1 mice had a significantly thicker photoreceptor ONL, IS and RPE and thinner OS compared to WT mice (Fig. 4 and Supplementary Table 2). The remainder of the retinal layers showed no significant changes, apart from a significant increase in OPL thickness at 12 weeks PNA and a significant decrease of the RNFL at 16 weeks PNA (Fig. 4 and Supplementary Table 2).

All evaluated l-DOPA treatment dosages partly normalised ONL thickness measurements obtained in OCA1 mice, with thickness values close to those observed for untreated WT mice throughout the experiment (Fig. 4 and Supplementary Table 2). Likewise, the decreased thickness of the OS was partly normalised by all doses of L-DOPA treatments (Fig. 4 and Supplementary Table 2).

OCA1 mice treated with l-DOPA at 12.3 mg/kg showed no statistical difference in RPE thickness compared to WT mice, suggesting a partial normalisation of this layer (Fig. 4 and Supplementary Table 2).

However, the differences observed in IS thickness measurements in untreated OCA1 mice were not corrected by l-DOPA treatment (Fig. 4 and Supplementary Table 2).

### l-DOPA normalises temporal changes in INL thickness of OCA1 mice

The retinal layer thickness differences observed in untreated OCA1 mice appeared early in development, being present from five weeks PNA, and persisted for the entire time course of the experiment, until 16 weeks PNA (Fig. 4 and Supplementary Table 2). Interestingly, throughout the study, OCA1 mice did not demonstrate the same tendency towards increasing RNFL or decreasing OPL thickness over time, as was observed in WT mice (Fig. 4 and Supplementary Table 2). The constant increase in IS thickness observed previously in l-DOPA-treated WT mice was also observed in untreated OCA1 mice as well as, partly, in OCA1 mice treated with l-DOPA at 6.15 and 12.3 mg/kg (Fig. 4 and Supplementary Table 2). Treatment with l-DOPA (6.15 and 9.35 mg/kg) restored the temporal decrease of INL thickness that was absent in untreated OCA1 mice (Fig. 4 and Supplementary Table 2). In addition, a temporal decrease in ONL thickness from 4 to 12 weeks PNA was observed in l-DOPA treated OCA1 mice, resulting in thickness values that were not significantly different to WT values (Fig. 4 and Supplementary Table 2).

### l-DOPA normalises changes in ONL cell distribution in OCA1 mice

**Figure 5:**
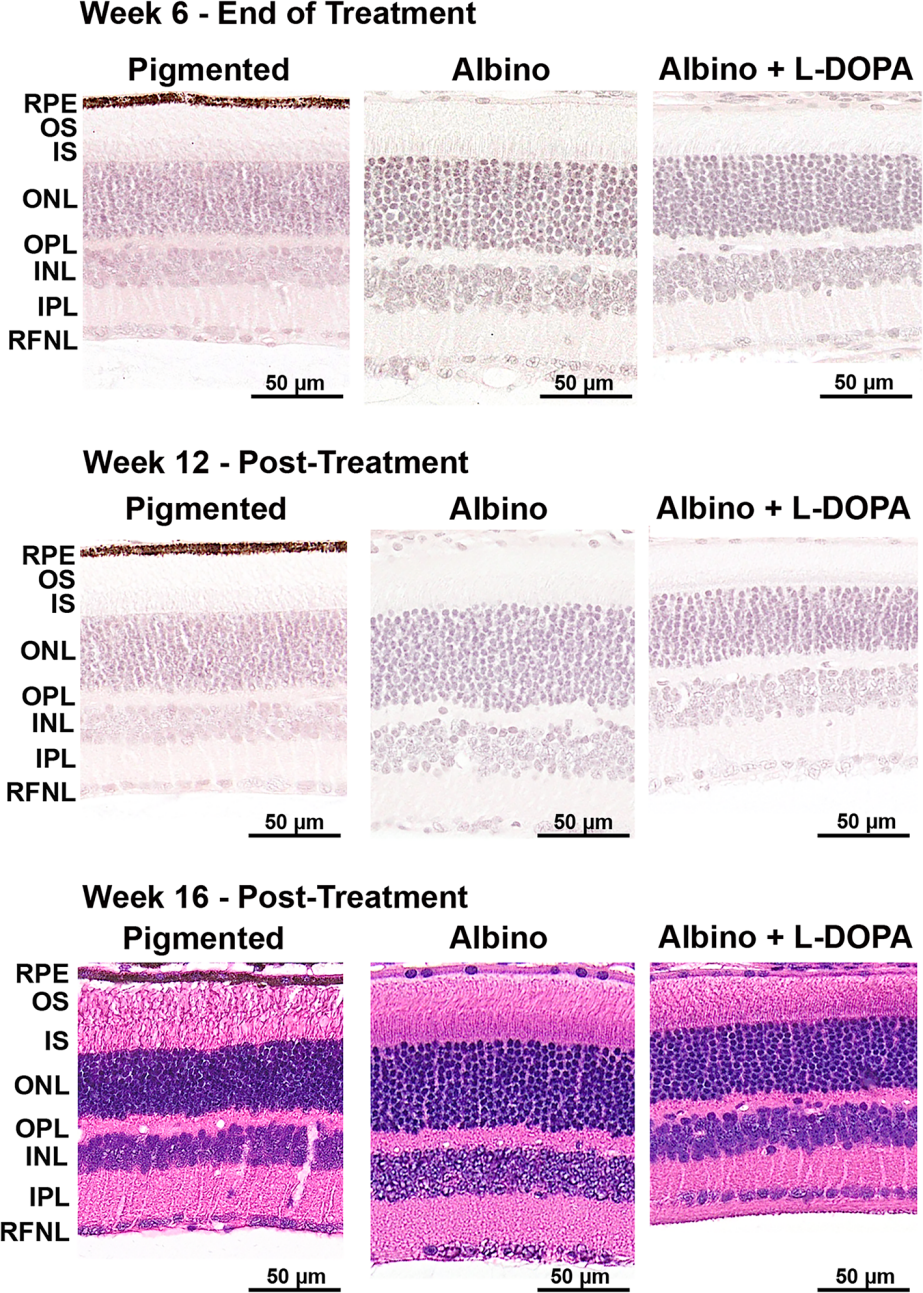
l-DOPA effect on ONL morphology in OCA1 mice. Retinal histological sections of OCA1 mice throughout the experiments stained with haematoxylin and eosin. OCA1 mice showed a thicker outer nuclear layer (ONL) compared with WT mice. In addition, the ONL had bigger cells and bigger spaces between cells. l-DOPA treatment corrected these anomalies. In the figure, we illustrate the results obtained with l-DOPA at 9.35 mg/kg, as an example. All l-DOPA doses had the same effect on ONL thickness. The INL of OCA1 mice also had bigger cells and spaces between cells when compared to WT mice. This was also corrected by l-DOPA treatment. *RPE* retinal-pigmented epithelium, *OS* outer segment of the photoreceptors, *IS* inner segment of the photoreceptors, *ONL* outer nuclear layer, *OPL* outer plexiform layer, *INL* inner nuclear layer, *IPL* inner plexiform layer, *RNFL* retinal nerve fibres layer. Scale bar = 50.

**Figure 6:**
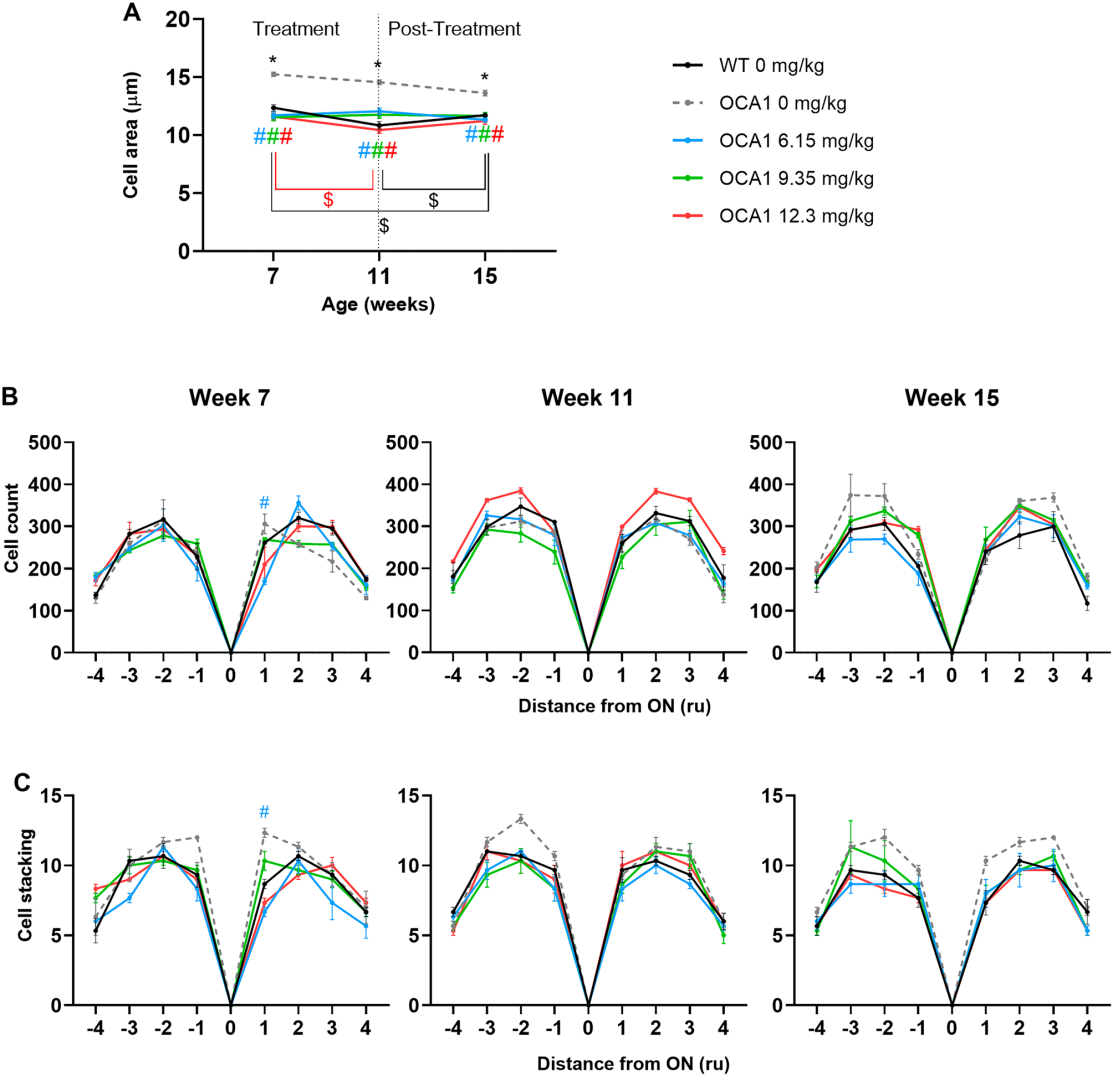
l-DOPA effect on ONL cell count in OCA1 mice. (**A**) ONL cell area measured in 50 random cells across the retina. Cells from non-treated OCA1 mice showed an increase in size compared with non-treated WT mice. All l-DOPA doses studied corrected this anomaly in size, returning cells to their normal areas. (**B**) Cells were counted in different locations in the ONL. According to its distance to the optic nerve (ON), cells were counted in four regions in the inferior side of the retina (sections – 1 to − 4, from closest to furthest to the ON) and in four regions in the superior side of the retina (sections 1 to 4, from closest to furthest to the ON), following the diagram in Fig. 1. (**C**) Stacks of cells were counted in different locations in the ONL. Results are shown as mean ± SEM. Asterisks indicate significant differences (P < 0.05) between non-treated WT mice and the different OCA1 mice groups. The colour of the asterisks indicates the albino group (i.e. blue asterisk corresponds with OCA1 mice treated with l-DOPA at 6.15 mg/kg) that is statistically different from non-treated WT mice. Hashtags indicate significant differences (P < 0.05) between OCA1 mice when compared with untreated OCA1 mice. The colour of the hashtag indicates the albino group that is statistically different from untreated OCA1 mice. The dollar symbols indicate significant differences (P < 0.05) across time in the same group of mice indicated by the colour of the symbol.

The increase in ONL thickness observed in untreated OCA1 mice (by OCT) and its recovery with l-DOPA treatment was confirmed histologically by H&E staining (Fig. 5). Cell counts and area measurements were obtained to further analyse the results (Fig. 6 and Supplementary Tables 3, 4 and 5).

The histology showed that ONL cells of untreated OCA1 mice were distributed differently, with larger gaps between the stained nuclei and wider cell areas than in WT mice (Fig. 5) (Fig. 6A and Supplementary Table 3).

Although the number of cells counted and their stacking was not significantly different, there was a tendency in untreated OCA1 mice towards increased numbers of cells by 15 weeks PNA (Fig. 6B and Supplementary Table 4). Cell stacking was also different, with a greater number of rows counted throughout the experiment (Fig. 6C and Supplementary Table 5).

Interestingly, throughout the experiment, OCA1 mice treated with l-DOPA, at any dose, showed a similar stacking pattern to that found in untreated WT mice. This suggests that some reorganization of the nuclei is taking place in treated mice (Fig. 6C and Supplementary Table 5).

### l-DOPA improves ERG responses in OCA1 mice

**Figure 7:**
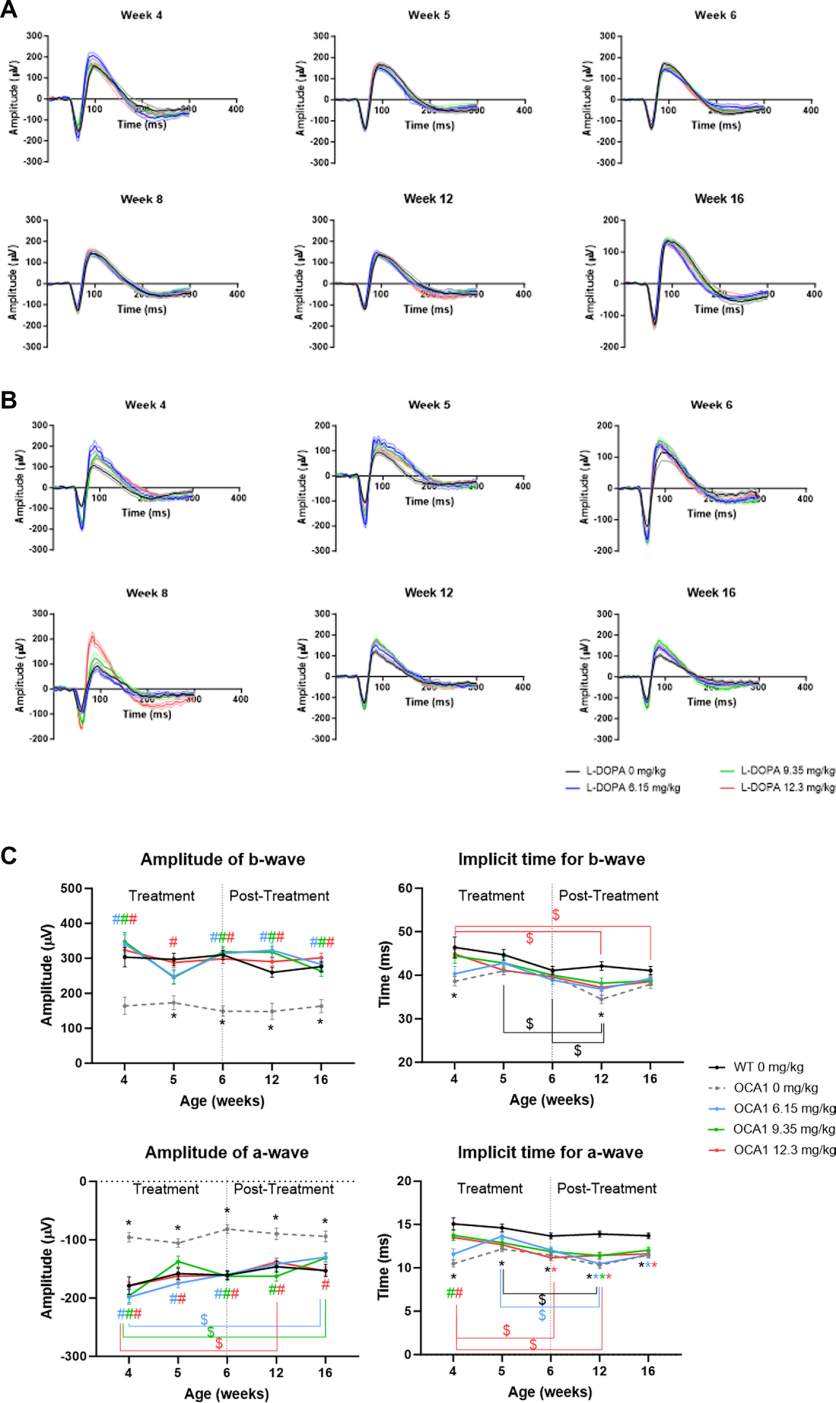
l-DOPA effect on retinal function in OCA1 mice. (**A**) ERG profiles obtained for WT mice throughout the experiment. Mice were exposed to a flash of light at time zero and the electrical response of the retina was recorded. The first decrease observed corresponds with the a-wave, a hyperpolarization caused mainly by activation of the photoreceptors. The subsequent depolarization (increase in µV) corresponds with activation of the inner retina, as part of the photoreceptor signal transduction process. Additional information regarding WT ERG results is included in Supplementary Table 6. (**B**). ERG profiles obtained for OCA1 mice throughout the experiment. Mice were exposed to a flash of light at time zero and the electrical response of the retina was recorded. The first decrease observed corresponds with the a-wave, a hyperpolarization caused mainly by activation of the photoreceptors. The subsequent depolarization (increase in µV) corresponds with activation of the inner retina, as part of the photoreceptor signal transduction process. Additional information regarding the ERG results obtained from OCA1 mice is included in Supplementary Table 6 (**C**). Retinal response to a light stimulus in OCA1 mice during the experiment including a- and b-wave amplitudes (left panels) and a- and b- wave implicit times (right panels) measured by ERG. OCA1 mice showed a dramatic decrease in a- and b-wave amplitudes and the implicit time of a-wave throughout the experiment. All different doses of l-DOPA tested rescued both a- and b-wave amplitudes to the normal values observed in WT mice. l-DOPA’s effect on a-wave was inconsistent over time with isolated improvements in different weeks with different doses (i.e. improvement in weeks 4 and 5 with all doses, in week 6 with l-DOPA at 6.15 and 9.35 mg/kg and in week 16 with L-DOPA at 9.35 mg/kg). Results are shown as mean ± SEM. Asterisks indicate significant differences (P < 0.05) between non-treated WT mice and the different OCA1 mice groups. The colour of the asterisks indicates the albino group (i.e. blue asterisk corresponds with OCA1 mice treated with l-DOPA at 6.15 mg/kg) that is statistically different from non-treated WT mice. Hashtags indicate significant differences (P < 0.05) between OCA1 mice when compared with untreated OCA1 mice. The colour of the hashtag indicates the albino group that is statistically different from untreated OCA1 mice. The dollar symbols indicate significant differences (P < 0.05) across time in the same group of mice indicated by the colour of the symbol.

Both a-wave and b-wave amplitudes of the scotopic ERG, as well as the a-wave implicit time in OCA1 mice were significantly decreased in comparison to WT mice (Fig. 7, and Supplementary Table 6) at all the time points studied from 6 to 16 weeks PNA. The b-wave implicit time was also decreased in untreated OCA1 mice, with significant decreases at 4 and 12 weeks PNA (Fig. 7, and Supplementary Table 6).

All three l-DOPA doses resulted in improvements in some of the ERG parameters recorded from the treated OCA1 mice (Fig. 7, and Supplementary Table 6). Specifically, there was a complete recovery of the a- and b-wave amplitudes that was observed from 4 weeks PNA and was sustained up to the final assessment at 16 weeks PNA, reaching higher amplitudes than in WT mice (Fig. 7; left panels, and Supplementary Table 6). The b-wave implicit time was similar in WT and OCA1, with slightly lower values for OCA1 mice that was only significantly different at 4 and 12 weeks PNA, and that was corrected with the three different doses of l-DOPA (Fig. 7; right panels, , and Supplementary Table 6). The a-wave implicit time appeared significantly decreased in OCA1 mice throughout the study and even though there was a significant recovery to WT values in OCA1 mice treated with l-DOPA for the duration of treatment, this effect was not sustained following completion of l-DOPA treatment at 6 weeks PNA (Fig. 7; right panels, and Supplementary Table 6).

Similar to WT mice, there was no change over time in the amplitude of b-wave, whereas a significant decrease in a-wave amplitude was observed in l-DOPA-treated OCA1 mice, suggesting a possible decrease in the action of l-DOPA over time (Fig. 7; left panels, and Supplementary Table 6). The a- and b- waves implicit times also showed a significant decrease over time in both untreated and l-DOPA-treated OCA1 mice (Fig. 7; right panels, and Supplementary Table 6).

### l-DOPA improves spatial frequency thresholds in OCA1 mice

**Figure 8:**
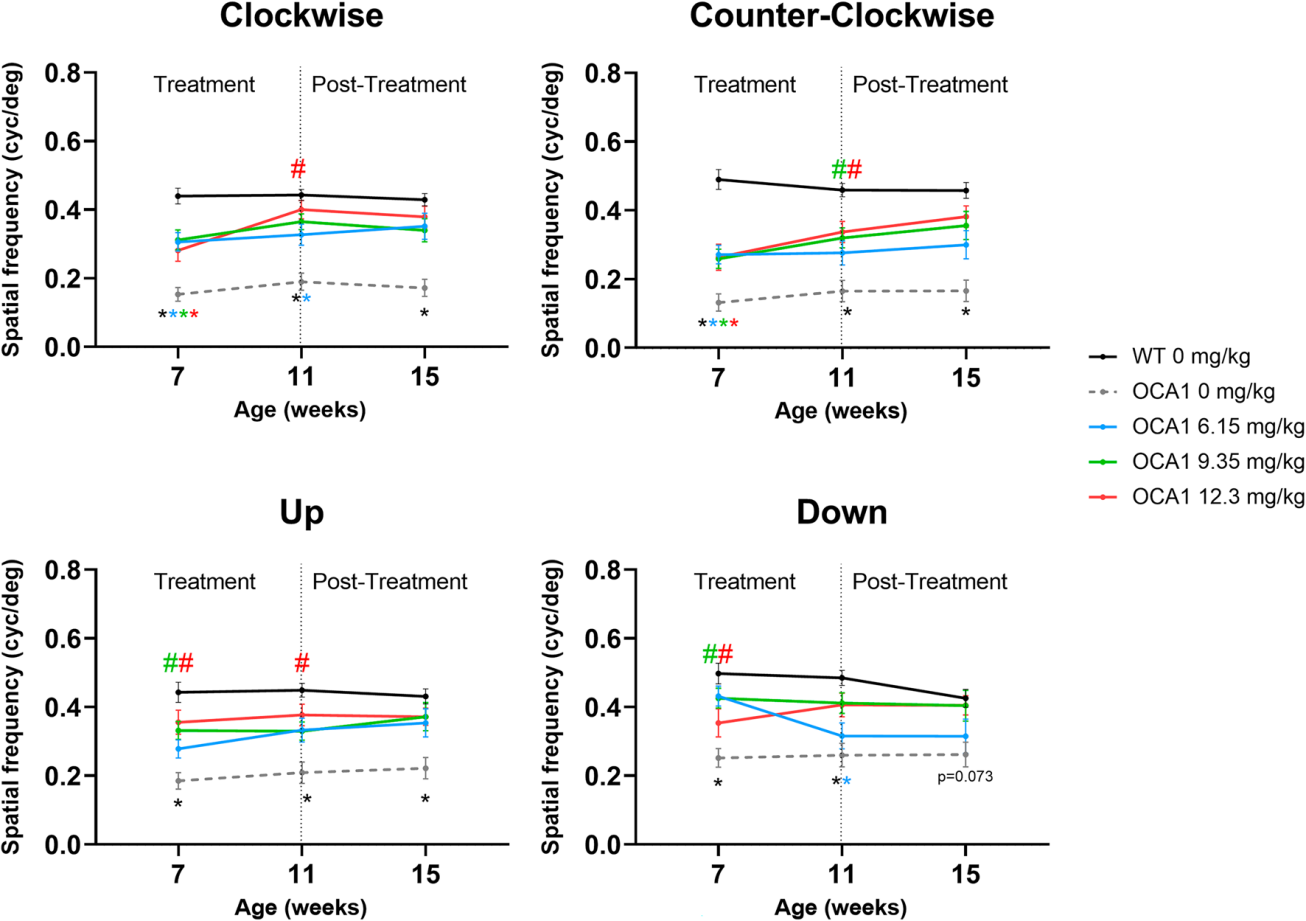
l-DOPA effects on spatial frequency thresholds in OCA1 mice. Spatial frequency thresholds were calculated for the four directions (clockwise, counter clockwise, up and down). In all cases, untreated OCA1 mice exhibited a significant decrease in the thresholds that was rescued by l-DOPA. Results are shown as mean ± SEM. Asterisks indicate significant differences (P < 0.05) between non-treated WT mice and the different OCA1 mice groups. The colour of the asterisks indicates the albino group (i.e. blue asterisk corresponds with OCA1 mice treated with L-DOPA at 6.15 mg/kg) that is statistically different from non-treated WT mice. Hashtags indicate significant differences (P < 0.05) between OCA1 mice when compared with untreated OCA1 mice. The colour of the hashtag indicates the albino group that is statistically different from untreated OCA1 mice. The dollar symbols indicate significant differences (P < 0.05) across time in the same group of mice indicated by the colour of the symbol.

OCA1 mice showed reduced spatial frequency thresholds in all the directions studied (Fig. 8 and Supplementary Table 7), in comparison to WT mice at the three time points assessed.

All three doses of l-DOPA resulted in improvements in the spatial frequency thresholds recorded from treated OCA1 mice. Specifically, the clockwise and counter clockwise directions showed a partial recovery of the spatial frequency thresholds that were observed from 11 weeks PNA. Partial recovery of normal threshold levels for vertical directions was observed from 7 weeks PNA, with no statistical differences evident in comparison to either WT or OCA1 control mice (Fig. 8 and Supplementary Table 7).

There were no significant changes in the spatial frequency thresholds recorded throughout the different time points of the study, which maintained stable values from 7 to 15 weeks PNA (Fig. 8 and Supplementary Table 7).

### A minimal l-DOPA treatment duration of 28 days is necessary for sustained improvements of retinal function in OCA1 mice

The improvements in retinal function previously observed in OCA1 mice treated with l-DOPA 12.3 mg/kg were replicated for treatment durations of 12, 28 and 56 days. However, sustained improvements following completion of treatment could only be achieved following 28 and 56 days of treatment (Table 2).

### l-DOPA treatment upregulates ocular PEDF expression in OCA1 mice

**Figure 9:**
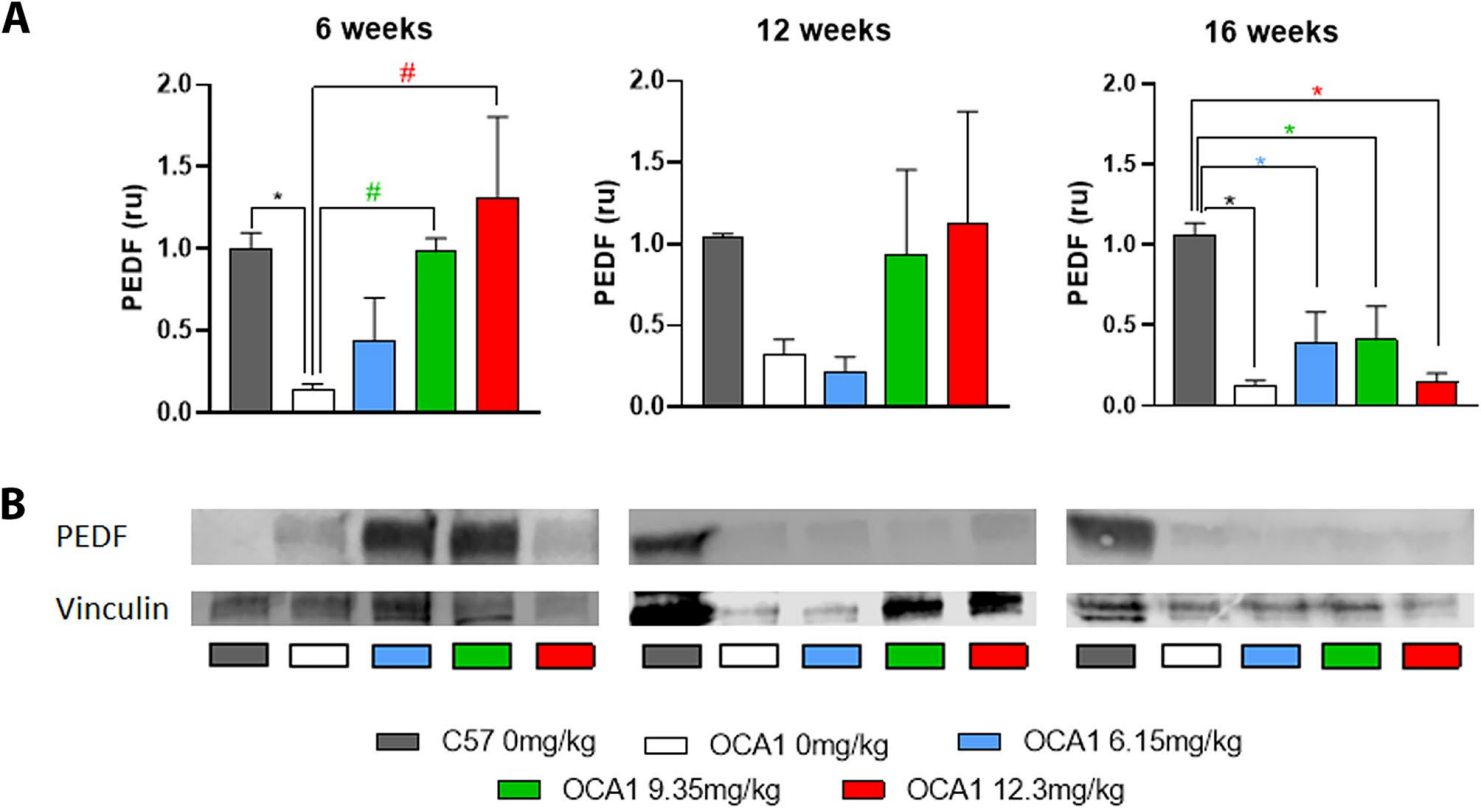
PEDF level in untreated C57 and OCA1 mice and OCA1 mice treated with l-DOPA during retinal development. Densitometry analysis of western blot results for PEDF levels. The PEDF levels were normalised to the loading control Vinculin, with the loading control considered to be 1 in all experiments. The normalised PEDF levels for OCA1 mice were calculated as a fold change of the expression level in C57 mice. Data is shown as mean ± SEM of 4 independent experiments. Representative images of the respective bands for PEDF (top panel) and vinculin (bottom panel) are shown. Two-way ANOVAs were performed and *indicates statistically significant differences (p < 0.05) between OCA1 and C57 mice. #shows statistically significant differences (p < 0.05) between control untreated OCA1 mice and l-DOPA treated OCA1 mice.

Western blot analysis of ocular PEDF levels demonstrated that untreated OCA1 mice had significantly reduced ocular PEDF levels in comparison to WT mice. l-DOPA treatment resulted in a dose-dependent increase in ocular PEDF levels in OCA1 mice, with the 6.15 and 12.3 mg/kg doses restoring PEDF expression to levels comparable to those seen in WT mice (Fig. 9 and Supplementary Table 8).

### l-DOPA supplementation modulates neurogenesis and synaptogenesis in OCA1 mice

**Figure 10:**
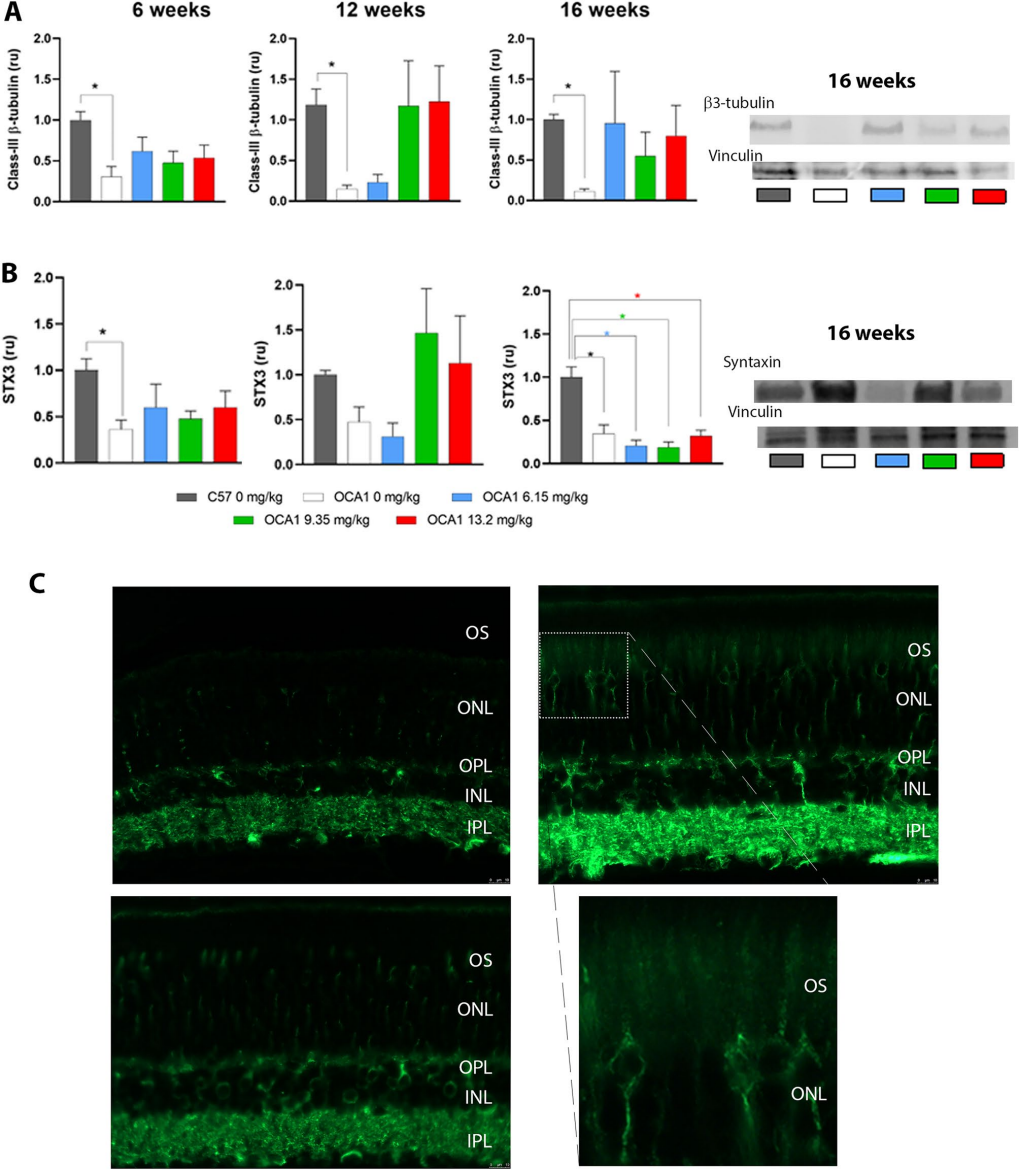
Class III β-tubulin and syntaxin 3 levels in untreated C57 and OCA1 mice and OCA1 mice treated with l-DOPA during retinal development. Densitometry analysis of Western blot results for class-III β-tubulin (**A**) and syntaxin (**B**) levels. The class-III β -tubulin and syntaxin were normalised to the Vinculin loading control, with Vinculin considered to be 1 in all experiments. The normalised levels of class-III β-tubulin and syntaxin were calculated as a fold change of the expression level in C57 mice. Data is shown as mean ± SEM of 4 independent experiments. Two-way ANOVAs were performed and * indicates a significant difference between OCA1 and C57 mice (p < 0.05). Representative western blots for both antibodies at 16 weeks are shown. (**C**) A comparison of class-III β-tubulin expression in C57, untreated OCA1 mice and OCA1 mice treated with 12.3 mg/kg l-DOPA. There is abnormal localisation of class-III β-tubulin to the OS of untreated OCA1 mice (white arrow). A zoomed in area of this is shown. This expression of class-III β-tubulin is not evident in l-DOPA treated OCA1 mice. Scale bar represents 10um. *STX3* syntaxin 3, *OS* photoreceptor outer segments, *ONL* outer nuclear layer, *OPL* outer plexiform layer, *INL* inner nuclear layer, *IPL* inner plexiform layer.

At all ages analysed, OCA1 mice showed significantly decreased levels of both class-III β-tubulin and syntaxin 3 measured by western blot (Fig. 10A and B respectively and Supplementary Table 8). These levels were partly restored in OCA1 mice when treated with l-DOPA. The restoration was maintained for the full 10-week post-treatment follow up period in the case of β3-tubulin but was lost for syntaxin 3.

In addition, confocal immunofluorescence studies identified erroneous localisationof class-III β-tubulin to the outer segments of the photoreceptors in OCA1 mice (Fig. 10C). High doses (12.3 mg/kg) of l-DOPA treatment were able to restore the correct retinal distribution of those markers.

## Discussion

In this study, we aimed to define the optimal l-DOPA treatment parameters to safely rescue visual function in albinism. We did not identify any detrimental or toxic effects in WT or OCA mice treated with l-DOPA. This contrasts with reports that l-DOPA can exert toxic effects on the RPE cells in vitro^39,40^. Tissue culture studies may not be directly comparable to in vivo l-DOPA treatment studies, as the l-DOPA concentrations used in vitro exceed the known peak physiological concentrations in the developing murine eye^24^. Consistent with this, a similar discrepancy has been reported with regards to potential toxicity to the substantia nigra from l-DOPA treatment that was seen in vitro, but not identified in Parkinson’s patients treated with l-DOPA. Our results confirmed altered retinal morphology, impaired response to light stimulation and reduced visual acuity in untreated OCA1 mice compared with WT mice over a range of treatment parameters. OCA1 murine retinas showed a different distribution of the outer retinal layers, with an increased ONL, IS and RPE thickness and a reduced OS length. The differences observed in comparison to our previous results^14^ are, in part, due to a change in our OCT analysis technique, from a manual 24 point retinal segmentation system to an automated volumetric segmentation system, recently provided by the manufacturer.

The increase in ONL thickness found in the OCTs of untreated OCA1 mice was further analysed by histology, with several differences identified in its structure when compared with untreated WT mice. Firstly, the size of the nuclei in the ONL from OCA1 mice appeared bigger, with a disorganised stacking pattern and irregular spacing observed between the cells and stacks. This altered structural organisation of the layer may explain why it appeared thicker even when it contained less cells at week 6. These results are similar to results reported in another rodent model of albinism, the deer mouse, where the photoreceptor diameter in albino mice was larger than in the pigmented mice, resulting in a decreased density of cells per mm^2^^41^.

There was a marked increase in the number of nuclei present in the ONL at 12 and 16 weeks PNA, associated with only a slight increase in the thickness of the layer, suggesting a constant reorganization of the cells, suggesting some level of neuroretinal plasticity. Interestingly, the number of cells in the ONL in OCA1 mice was significantly lower compared to WT mice and, while the OCA1 mice showed an increase in the number of cells from 6 to 16 weeks PNA, there was no change in the cell count in WT mice. According to Young^26^, the process of cell differentiation actively continues in the mouse retina until PND 11, which is in accordance with the results obtained in WT mice in this study. However, the cell cycle is altered in albino rodents, resulting in a delay in retinal development^42,43^. Similarly, an altered cell cycle could explain the increase in cell count found in the ONL of the OCA1 mice.

OCA1 mice also demonstrated a change in the IS/OS ratio, with a thicker IS and a reduced OS. The IS membrane contains the ion exchangers and channels responsible for maintaining the resting membrane potential and determining the cellular response to light stimuli^44^, as well as dopamine receptors^45^, which are required for both the light response and regulation of disc shedding in the OS. In our experiment, untreated OCA1 mice had a reduced latency on ERG to light stimuli than WT mice, which may be attributable to the altered morphology of its photoreceptors, specifically, the increase in IS thickness.

Similarly, the morphological changes observed in the IS/OS and RPE of OCA1 mice, might be the reason for the impaired retinal response to light stimuli observed in OCA1 mice. The relationship between the OS and the RPE in retinal function is well established^46,47^. The RPE acts as an important support for photoreceptor cells, by protecting them from the toxicity of photo-oxidative products created during photo-transduction. The OS is renewed constantly to eliminate the accumulated photo-oxidative products, releasing vesicles that are phagocytosed by the RPE^48^. The IS are responsible for synthesizing the proteins required for OS renewal^44,49^. We have demonstrated an abnormal accumulation of rhodopsin in the OS of OCA1 mice compared with WT mice (unpublished own data). This result, together with the observation of a decreased thickness in the OS and increased thickness of the IS and RPE of untreated OCA1 mice, suggests possible abnormalities of disc shedding in the developing retina of OCA1 mice. These findings contrast with studies performed of disc shedding in BALB/c mice^50,51^, an albino mouse strain with a different mutation in the *tyrosinase* gene^17^. In these studies, albino mice did not present any major changes in disc shedding when compared with pigmented mice^50,51^. However, the experiments were performed in mature, adult mice, whilst our studies, were carried out in mice that were still actively undergoing retinal development, which has a different environment.

Regarding retinal function, there was a significant decrease in the amplitudes of a- and b-wave as well as a decrease in the latency time recorded from OCA1 mice. This effect on the amplitudes was previously reported in our laboratory^14^. As a-wave amplitude is mainly a measure of photoreceptor response to a light stimuli^52^ and b-wave corresponds with the response in the inner retina, including communication between rod- bipolar cells^53^, our results suggest that alterations in retinal connectivity in OCA1 mice occur in the inner and outer retina. These alterations appear to have deleterious consequences for visual acuity. Previous studies in mice suggested that the development of visual thresholds reaches maturation before PND 30^54^. Our first visual threshold assessments were performed at 7 weeks of age, when the system would be considered to be mature and defined. As expected, the results obtained at this assessment were sustained longitudinally at each subsequent assessment at weeks 11 and 15, in both untreated WT and OCA1 mice. We demonstrated that untreated OCA1 mice had spatial frequency thresholds that were halved in comparison to WT mice. These findings are consistent with those previously described for albino rats and mice, where the spatial frequency thresholds were also half of the values obtained for pigmented rats and mice^55,56^. Moreover, the OCA1 mice in our study had similar spatial frequency thresholds to those previously reported in BALB/c mice^56^, another albino strain without tyrosinase activity.

The lack of tyrosinase activity in OCA1 causes a lack of l-DOPA in the RPE throughout development^24^. Previous work carried out in our laboratory demonstrated that supplementation of l-DOPA in order to mimic its normal physiological peak during retinal development improved retinal morphology and function^14^. This suggests that l-DOPA supplementation during the critical period of residual neuroplasticity, could improve retinal development and visual function in children with OCA^14^. Interestingly, recent studies have also shown an improvement in other retinal conditions including Age-related Macular Degeneration when patients are treated with l-DOPA^57^, suggesting that l-DOPA may also have a role in slowing or preventing retinal degenerative conditions.

In this study, we selected HED that have been established for use in infants and young children diagnosed with infantile dystonia and amblyopia^28^ to determine its potential clinical value in the context of OCA1. Three different doses were selected to determine the optimal dose of l-DOPA that can maximally rescue visual function in albinism, in preparation for future human clinical trials. It has previously been reported that oral l-DOPA supplementation had no detrimental or toxic effects on WT or OCA1 mice^14^. Our results confirmed that treatment with HED of l-DOPA in WT mice had no significant side effects. We also confirmed that a minimum of 28 days of treatment was required in order to obtain a sustained improvement in visual function. It is worth considering that due to interspecies variation in metabolic rates and physiological processes which alters the pharmacokinetics, we cannot be certain how accurate our HED calculations are. We have calculated the HED of l-DOPA using an established allometric method, which attempts to account for differences in physiological time by considering the differences in body surface area^35^. In the future, it would be interesting to determine the maximum safe dose at which efficacy plateaus.

Regarding morphology, l-DOPA treatment was able to correct some of the defects observed in untreated OCA1 mice. Specifically, l-DOPA normalized ONL and OS layer thicknesses. Histologically, there with fewer gaps between cells in the ONL in l-DOPA treated OCA1 mice and ONL cell size was similar to that observed in WT mice, ONL cell counts also adjusted to physiological levels (WT mice cell count) more rapidly compared to untreated OCA1. These morphological changes in treated OCA1 mice are likely mediated through l-DOPA’s effects on the cell cycle^43^. However, l-DOPA was unable to restore IS and RPE thickness values to normal levels. This suggests that the timing of treatment (from PND 15) may be too late to normalize such layers, which are already differentiated^27,58^.

We have demonstrated that HED of l-DOPA rescues retinal function in treated OCA1 mice, restoring both a- and b-wave ERG amplitudes to normal levels and significantly improving OKN spatial frequency thresholds in comparison to untreated OCA1 mice. This is consistent with earlier results reported from our laboratory in OCA1 mice treated with a higher dose of l-DOPA from birth or PND 15^14^. These findings are also consistent with work carried out by Lavado et al.^59^, which showed that only l-DOPA or its metabolic derivatives are required for normal retinal development. Subsequent mechanistic work carried out by Lopez et al.^60^ outlined the role of l-DOPA as a neurotransmitter in the RPE, where it binds to the OA1 G-protein coupled receptor to upregulate PEDF secretion to regulate retinal development. Consistent with this pathway, we demonstrated significantly reduced ocular PEDF levels in untreated OCA1 mice, and ocular PEDF levels equivalent to those found in WT mice in l-DOPA treated OCA1 mice.

The timing of l-DOPA treatment coincides with the peak of retinal synaptogenesis^27^. Potentially, l-DOPA treatment improves visual function in OCA1 mice by modulating synaptogenesis and consequently retinal connectivity. To investigate this, we explored the retinal expression of two proteins: syntaxin 3 and class-III β- tubulin in WT, l-DOPA treated and untreated OCA1 mice.

Syntaxin 3 is a SNARE protein that is expressed in the ribbon synapses formed by photoreceptors and bipolar cells in the retina, which plays an essential role in neurotransmission^61,62^. We found that there is significantly reduced expression of syntaxin 3 in untreated OCA1 mice, and that l-DOPA treatment reversed this difference. This suggests that l-DOPA has a key role to play in modulating synaptogenesis during retinal development.

Class-III β-tubulin is a constituent of neuronal microtubules and is essential for neuronal development^63^. In the developing retina, class-III β-tubulin is known to be expressed in immature neurons^64^. Class-III β-tubulin immunoreactivity normally increases as the retina matures, and is primarily expressed in ganglion cells^65^. We found that there is significantly reduced expression of class-III β-tubulin in untreated OCA1 mice, and that l-DOPA treatment reversed this effect. Interestingly, we also identified an abnormal distribution of class-III β-tubulin to the photoreceptor OS of OCA1 mice. This could potentially reflect the dynamic instability associated with the cell cycle abnormalities that are known to occur during neurogenesis of the albino retina^43^, suggesting an important role for l-DOPA in modulating retinal neurogenesis.

In preparation for future clinical trials, we have demonstrated that treatment with HED of l-DOPA that are already in established use for children with infantile dystonia and amblyopia from 15 days PNA, can rescue retinal morphology and visual function in OCA1 mice. Sustained improvements of visual function can only be obtained with a minimal of 28 days of treatment, coinciding with the normal peak and duration of ocular l-DOPA levels and the timing of retinal synaptogenesis. We have also confirmed that l-DOPA treatment upregulates PEDF expression and achieves improvements in visual function by modulating retinal synaptogenesis. Further work is needed to fully interrogate the effects of l-DOPA on retinal synaptogenesis and identify other potential therapeutic targets that can prevent visual impairment in infants and young children with albinism.

### Supplementary Information

Supplementary Legends.

Supplementary Figure 1.

Supplementary Figure 2.

Supplementary Figure 3.

Supplementary Figure 4.

Supplementary Figure 5.

Supplementary Figure 6.

Supplementary Table 1.

Supplementary Table 2.

Supplementary Table 3.

Supplementary Table 4.

Supplementary Table 5.

Supplementary Table 6.

Supplementary Table 7.

Supplementary Table 8.

## Data Availability

The datasets generated and/or analysed during this study are openly available from the University of Southampton repository at 10.5258/SOTON/D2608.

## Abbreviations

ERG: Electroretinogram
H&E: Haematoxylin/eosin
INL: Inner nuclear layer
IPL: Inner plexiform layer
IS: Inner segment of the photoreceptors
OCA: Oculocutaneous albinism
OCA1: Oculocutaneous albinism type 1
OCT: Optical coherence tomography
OPL: Outer plexiform layer
ON: Optic nerve
ONL: Outer nuclear layer of the photoreceptors
OS: Outer segment of the photoreceptors
PNA: Postnatal age
PND: Postnatal day
RNFL: Retinal nerve fibre layer
RPE: Retinal-pigmented epithelium
WT: Wild type
