# Supplementary material for: Human equivalent doses of l-DOPA rescues retinal morphology and visual function in a murine model of albinism
Source: Sci Rep. 2023 Oct 11;13:17173. doi: 10.1038/s41598-023-44373-3 (PMC10567794; doi:10.1038/s41598-023-44373-3)
Supplement: Supplementary file 1 — Supplementary Legends. [file 41598_2023_44373_MOESM1_ESM.docx]

**Supplementary Figure 1.** Body weight evolution in both WT and OCA1 mice throughout the experiment. Data are expressed as mean ± SEM. Letters increase following a significant increase in weight between timepoints (i.e. black a increasing to b at between 4 and 5 weeks shows a significant difference in weight but stays at b between 5 and 6 weeks when no significant increase in weight was seen).

**Supplementary Figure 2**. Sex differences in the retinal layers thickness in untreated and L-DOPA-treated WT mice in weeks 6, 12 and 16. Data are expressed as mean ± SEM. No statistically significant differences were found.

**Supplementary Figure 3**. Sex differences in the retinal layers thickness in untreated and L-DOPA-treated OCA1 mice in weeks 6, 12 and 16. Data are expressed as mean ± SEM. No statistically significant differences were found.

**Supplementary Figure 4**. Sex differences in the electrical response of the retina to a light stimulus in untreated and L-DOPA-treated WT and OCA1 mice in weeks 6, 12 and 16. Amplitudes and implicit times of a- and b-wave were measured. Data are expressed as mean ± SEM. No statistically significant differences were found.

**Supplementary Figure 5**. Sex differences in the spatial frequency thresholds measured in un treated and L-DOPA-treated WT and OCA1 mice in weeks 6, 12 and 16. The four directions of movement were measured (clockwise, counter clockwise, up and down). Data are expressed as mean ± SEM. No statistically significant differences were found.

**Supplementary Figure 6.** Full western blots of trimmed blots used in the manuscript. The bands of interest are highlighted in a red box where the Vinculin control is always the highest box on the gel and the band of interest below. The lanes of interest are also labelled.

**Supplementary Table 1. Results of statistical analyses.** The different variables studied (retinal layers thickness obtained by OCT; cell count, stacking and area obtained by histological analyses; b-wave and a-wave amplitudes and implicit times measured by ERG; and spatial frequency thresholds measured by OptoMotry) were analysed using general linear mixed modelling in SPSS with “genotype”, “age” and “treatment” as fixed factors, followed by a Bonferroni post-hoc test. P-values obtained for the linear model are detailed. Asterisks indicate significant differences (P<0.05) in the specific category.

**Supplementary Table 2.** Retinal layers thickness values untreated and L-DOPA-treated WT and OCA1 mice throughout the experiment. Results are shown as mean ± SD. Numbers per group are indicated in *italics*. Asterisks indicate significant differences (P<0.05) between untreated WT mice and other groups. Hashtags indicate significant differences (P<0.05) between OCA1 mice when compared with untreated OCA1 mice. The dollar symbols indicate significant differences (P<0.05) across time in the same group of mice. The colour code indicates, in each category, in red the values closer to untreated WT mice values and in blue the values closer to untreated OCA1 mice values.

**Supplementary Table 3.** Cell area values for untreated and L-DOPA-treated WT and OCA1 mice throughout the experiment. Results are shown as mean ± SD. Numbers per group are indicated in *italics*. Asterisks indicate significant differences (P<0.05) between untreated WT mice and the other groups. Hashtags indicate significant differences (P<0.05) between OCA1 mice when compared with untreated OCA1 mice. The dollar symbols indicate significant differences (P<0.05) across time in the same group of mice. The colour code indicates, in each category, in red the values closer to untreated WT mice values and in blue the values closer to untreated OCA1 mice values.

**Supplementary Table 4.** Cell count values for untreated and L-DOPA-treated WT and OCA1 mice throughout the experiment. The numbers of the positions indicate the location of the cells in the retina relative to the optic nerve (ON) in the inferior retina (sections -1 to -4, from closest to furthest to the ON, central and peripheral retina, respectively) and in the superior retina (sections 1 to 4, from closest to furthest to the ON, central and peripheral retina, respectively) as illustrated in Figure 1. Results are shown as mean ± SD. Numbers per group are indicated in *italics*. Asterisks indicate significant differences (P<0.05) between untreated WT mice and the other groups. Hashtags indicate significant differences (P<0.05) between OCA1 mice when compared with untreated OCA1 mice. The dollar symbols indicate significant differences (P<0.05) across time in the same group of mice. The colour code indicates, in each category, in red the values closer to untreated WT mice values and in blue the values closer to untreated OCA1 mice values.

**Supplementary Table 5.** Cell stacking values for untreated and L-DOPA-treated WT and OCA1 mice along the experiment. The numbers of the positions indicate the location of the cells in the retina relatively to the optic nerve (ON) in the inferior retina (sections -1 to -4, from closest to furthest to the ON, central and peripheral retina, respectively) and in the superior retina (sections 1 to 4, from closest to furthest to the ON, central and peripheral retina, respectively), as illustrated in Figure 1. Results are shown as mean ± SD. Numbers per group are indicated in *italics*. Asterisks indicate significant differences (P<0.05) between untreated WT mice and the other groups. Hashtags indicate significant differences (P<0.05) between OCA1 mice when compared with untreated OCA1 mice. The dollar symbols indicate significant differences (P<0.05) across time in the same group of mice. The colour code indicates, in each category, in red the values closer to untreated WT mice values and in blue the values closer to untreated OCA1 mice values.

**Supplementary Table 6.** ERG values for untreated and L-DOPA-treated WT and OCA1 mice throughout the experiment. Amplitudes and implicit times of a- and b-wave were measured. Results are shown as mean ± SD. Numbers per group are indicated in *italics*. Asterisks indicate significant differences (P<0.05) between untreated WT mice and the other groups. Hashtags indicate significant differences (P<0.05) between OCA1 mice when compared with untreated OCA1 mice. The dollar symbols indicate significant differences (P<0.05) across time in the same group of mice. The colour code indicates, in each category, in red the values closer to untreated WT mice values and in blue the values closer to untreated OCA1 mice values.

**Supplementary Table 7.** Spatial frequency threshold values for untreated and L-DOPA-treated WT and OCA1 mice throughout the experiment. The four directions of movement were measured (clockwise, counter clockwise, up and down). Results are shown as mean ± SD. Numbers per group are indicated in *italics*. Asterisks indicate significant differences (P<0.05) between non-treated WT mice and the rest of the groups. Hashtags indicate significant differences (P<0.05) between OCA1 mice when compared with untreated OCA1 mice. The dollar symbols indicate significant differences (P<0.05) across time in the same group of mice. The colour code indicates, in each category, in red the values closer to untreated WT mice values and in blue the values closer to untreated OCA1 mice values.
