# Supplementary figures and images for: Human equivalent doses of l-DOPA rescues retinal morphology and visual function in a murine model of albinism
Source: Sci Rep. 2023 Oct 11;13:17173. doi: 10.1038/s41598-023-44373-3 (PMC10567794; doi:10.1038/s41598-023-44373-3)

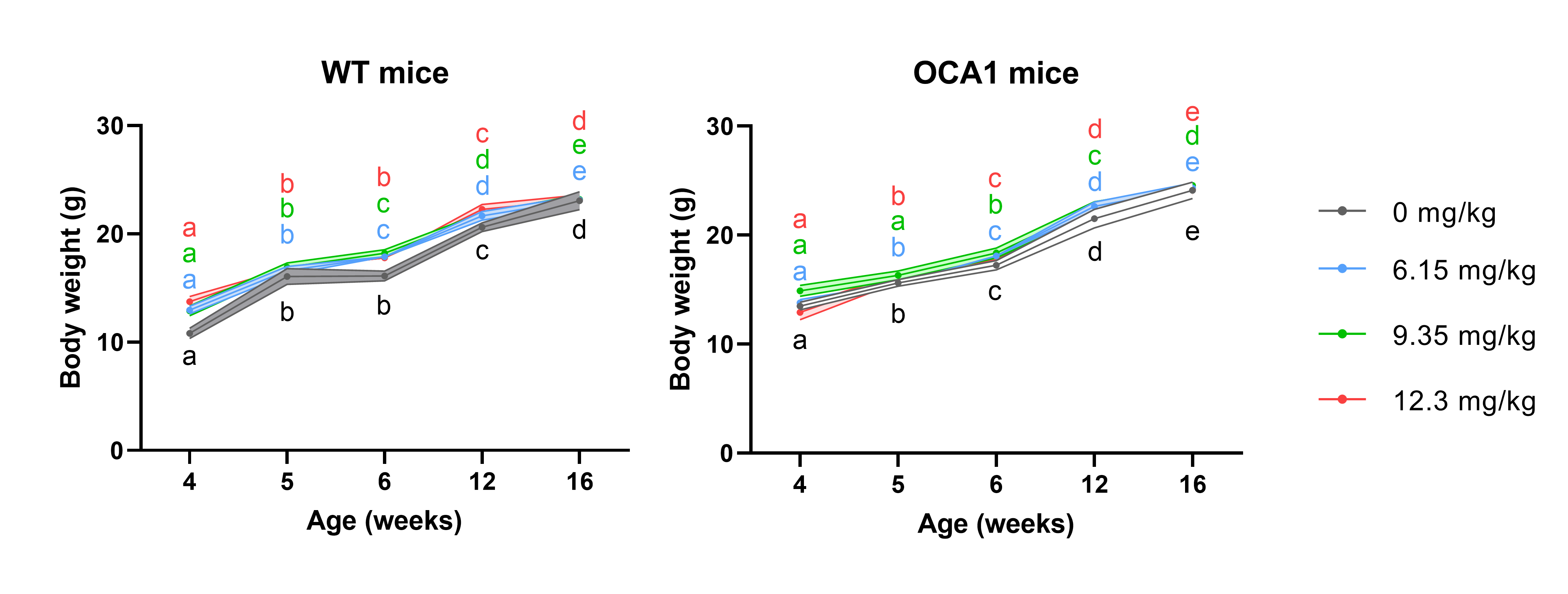

Supplement: Supplementary file 2 — Supplementary Figure 1. [file 41598_2023_44373_MOESM2_ESM.tif]

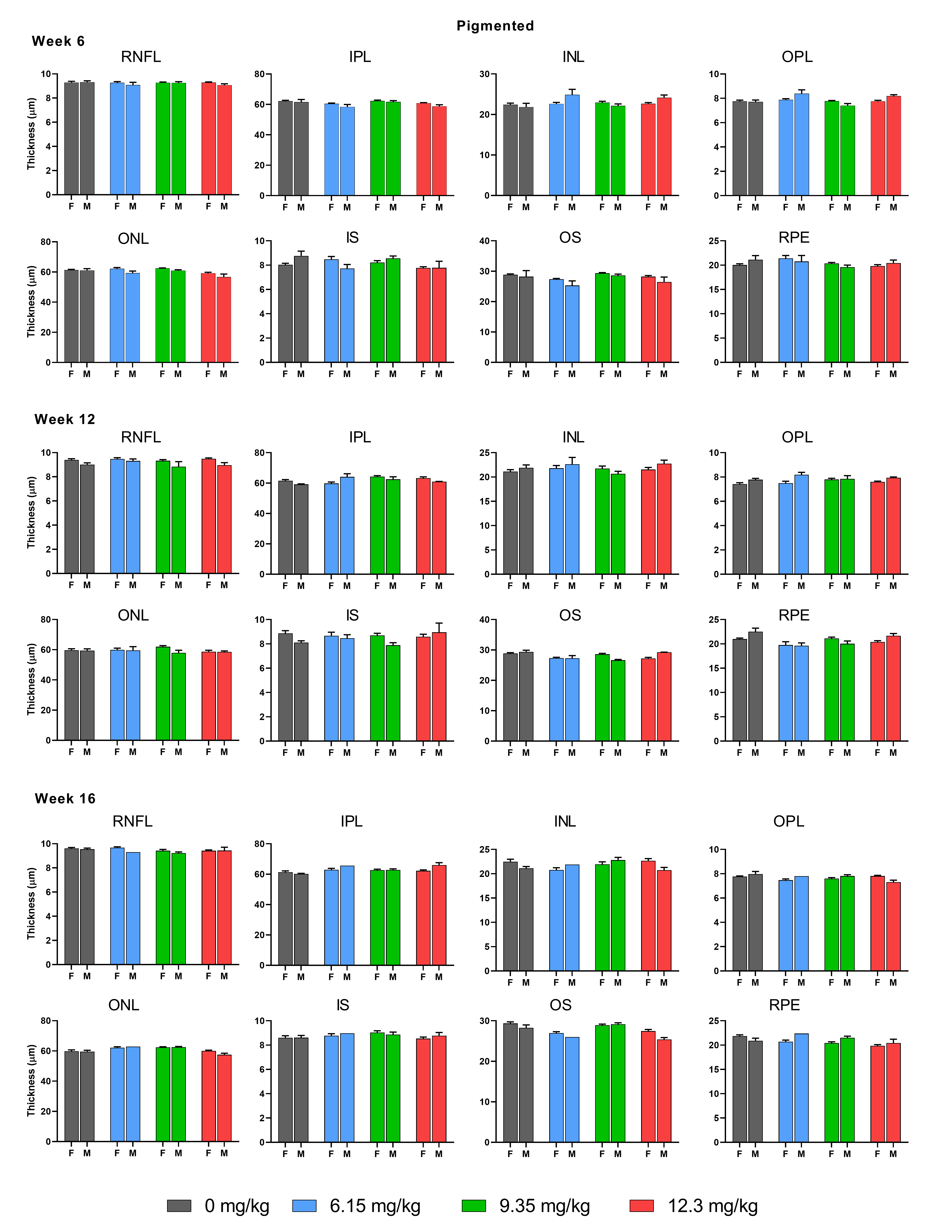

Supplement: Supplementary file 3 — Supplementary Figure 2. [file 41598_2023_44373_MOESM3_ESM.tif]

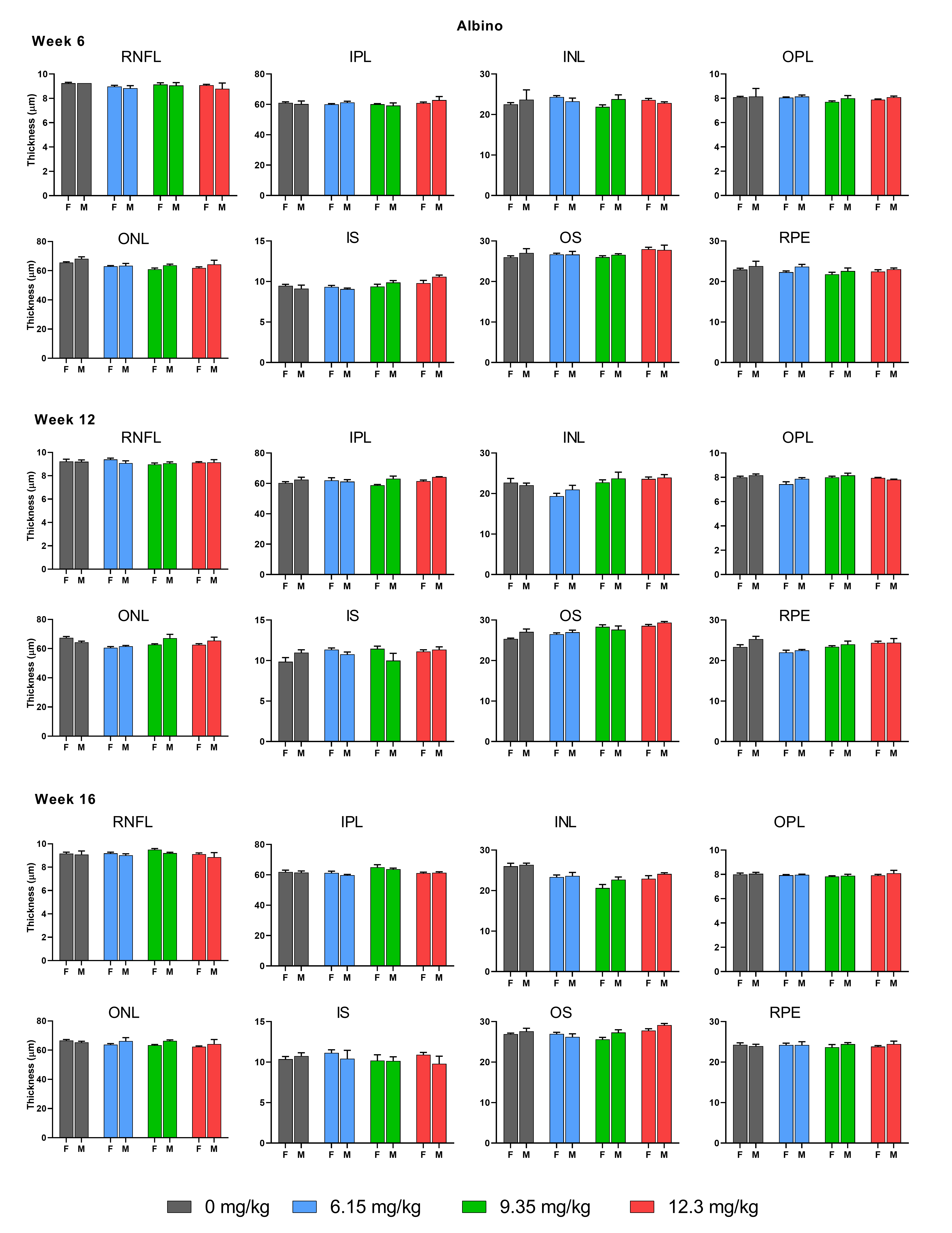

Supplement: Supplementary file 4 — Supplementary Figure 3. [file 41598_2023_44373_MOESM4_ESM.tif]

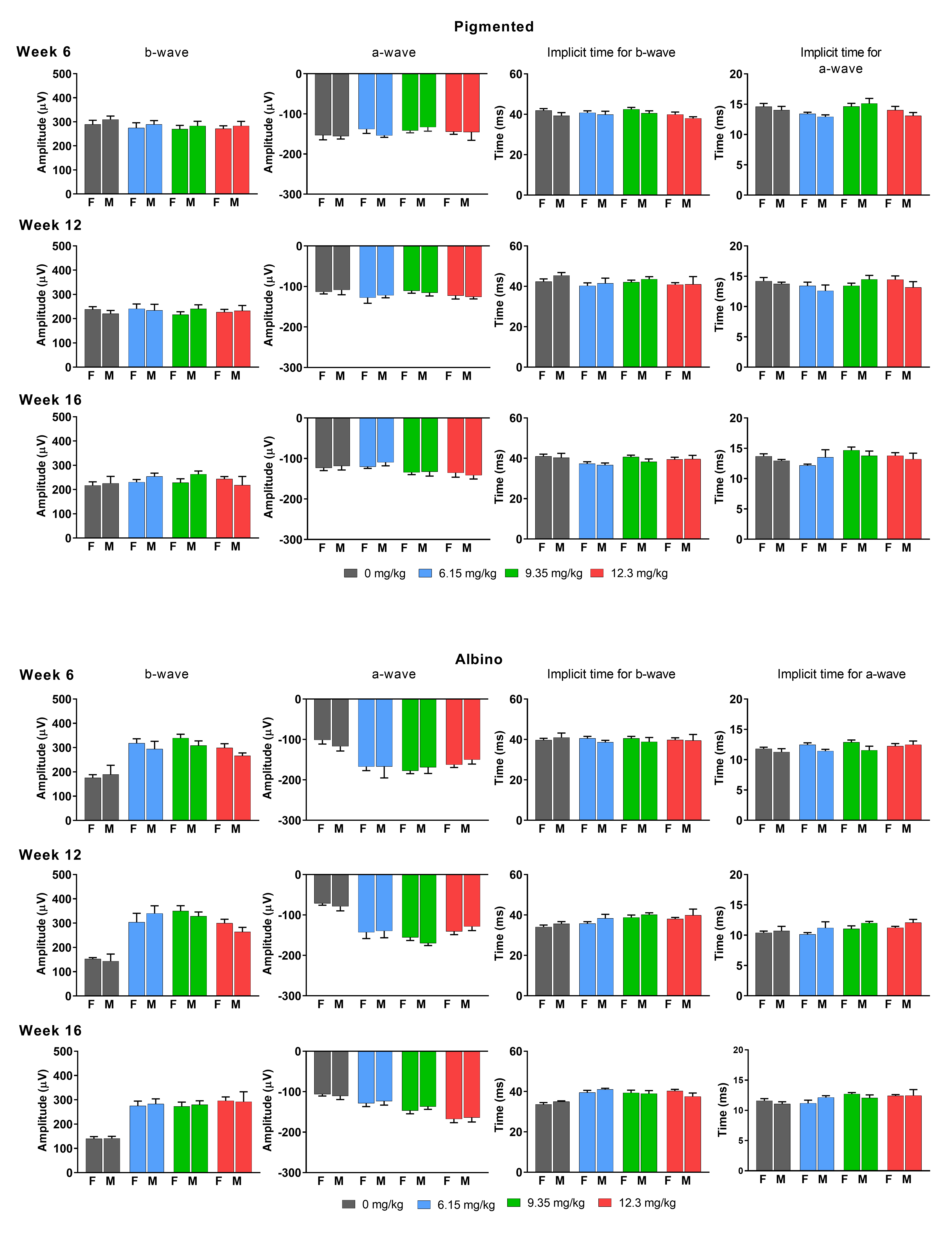

Supplement: Supplementary file 5 — Supplementary Figure 4. [file 41598_2023_44373_MOESM5_ESM.tif]

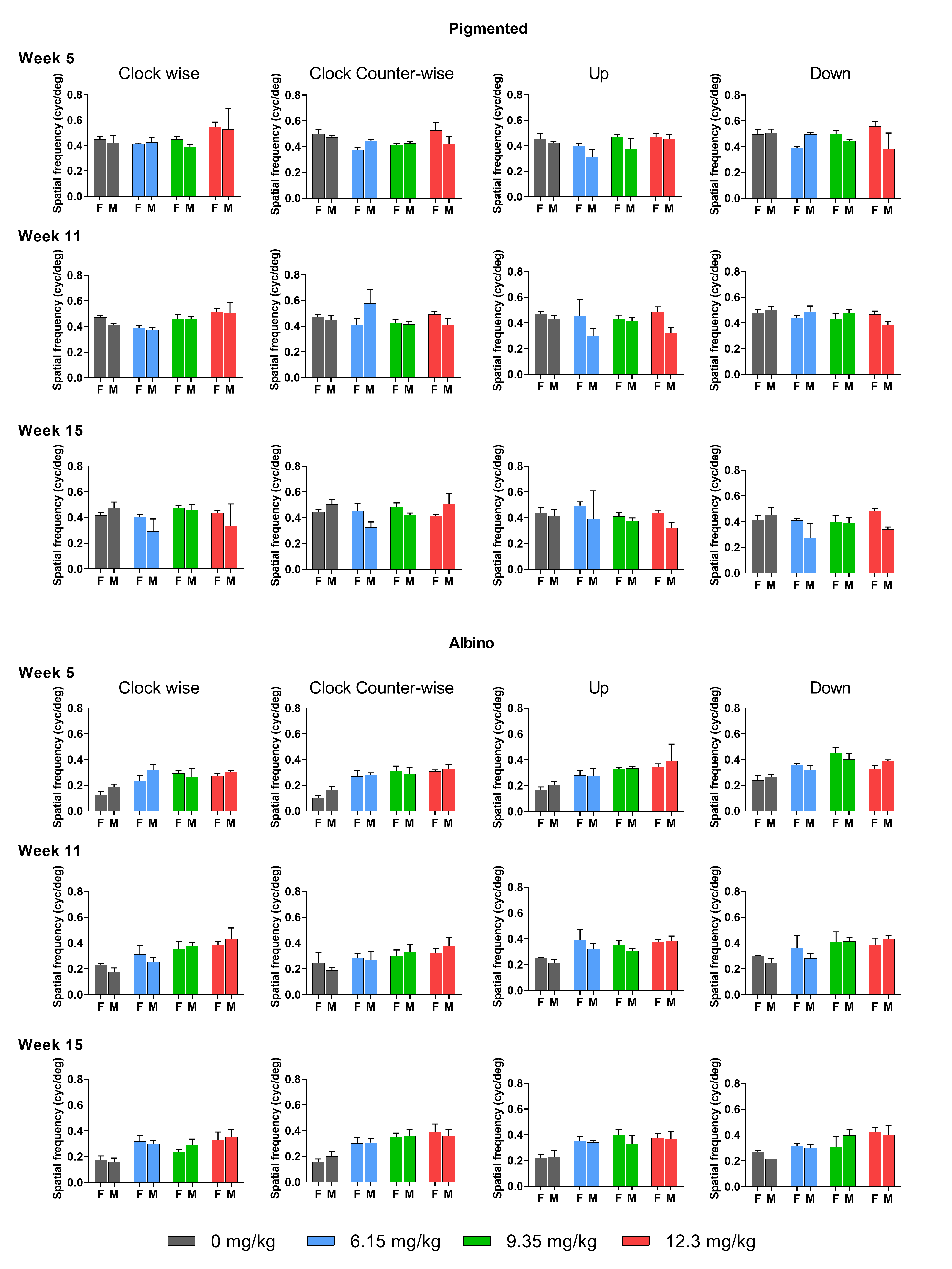

Supplement: Supplementary file 6 — Supplementary Figure 5. [file 41598_2023_44373_MOESM6_ESM.tif]
