# Supplementary material for: Human equivalent doses of l-DOPA rescues retinal morphology and visual function in a murine model of albinism
Source: Sci Rep. 2023 Oct 11;13:17173. doi: 10.1038/s41598-023-44373-3 (PMC10567794; doi:10.1038/s41598-023-44373-3)

6 weeks PEDF and Vinculin

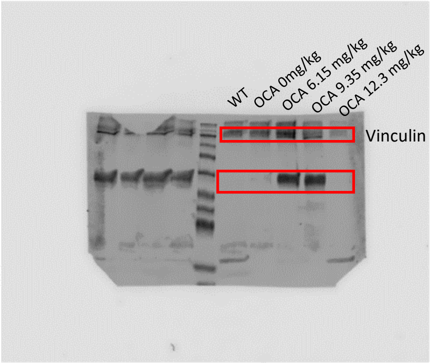

12 weeks PEDF and Vinculin

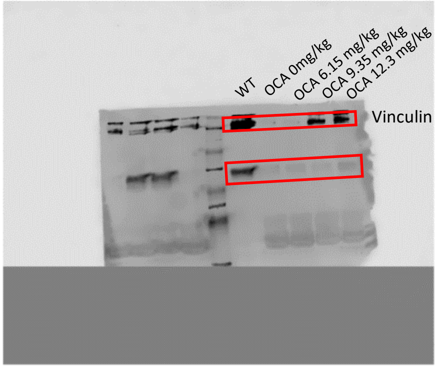

16 weeks PEDF blot 1 and Vinculin

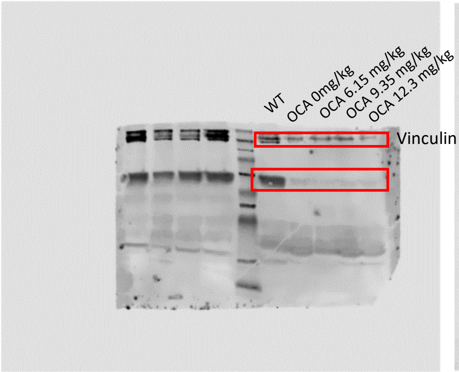

16 weeks PEDF blot 2 and Vinculin

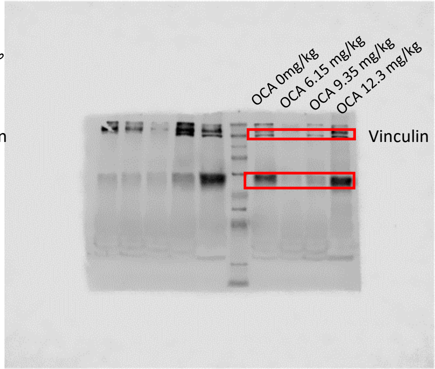

B3-tubulin and Vinculin

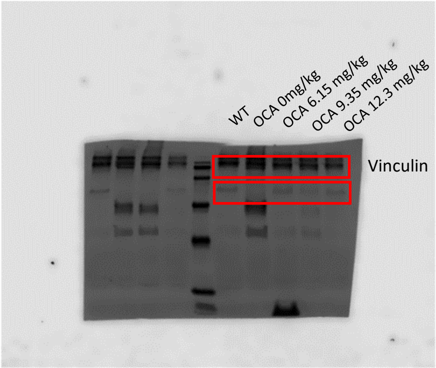

Syntaxin and Vinculin

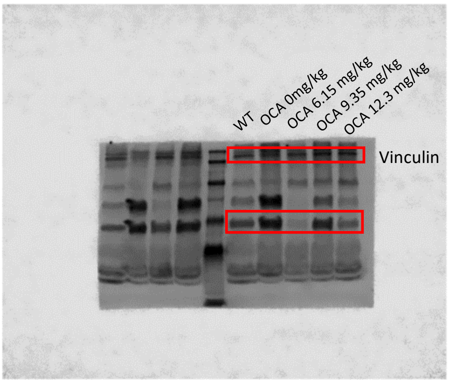

Supplement: Supplementary file 7 — Supplementary Figure 6. [file 41598_2023_44373_MOESM7_ESM.pdf]
