# Supplementary material for: Human equivalent doses of l-DOPA rescues retinal morphology and visual function in a murine model of albinism
Source: Sci Rep. 2023 Oct 11;13:17173. doi: 10.1038/s41598-023-44373-3 (PMC10567794; doi:10.1038/s41598-023-44373-3)
Supplement: Supplementary file 8 — Supplementary Table 1. [file 41598_2023_44373_MOESM8_ESM.pdf]

## Retinal morphology

| | Retinal layers thickness | | | | | | | |
|---|---|---|---|---|---|---|---|---|
| | RNFL | IPL | INL | OPL | ONL | IS | OS | RPE |
| Genotype | 0.000 * | 0.633 | 0.040 * | 0.022 * | 0.000 * | 0.000 * | 0.000 * | 0.000 * |
| Treatment | 0.313 | 0.000 * | 0.006 * | 0.423 | 0.000 * | 0.165 | 0.000 * | 0.003 * |
| Age | 0.000 * | 0.001 * | 0.000 * | 0.000 * | 0.000 * | 0.000 * | 0.298 | 0.000 * |
| Genotype * Treatment | 0.208 | 0.000 * | 0.518 | 0.361 | 0.000 * | 0.000 * | 0.000 * | 0.001 * |
| Genotype * Age | 0.003 * | 0.007 * | 0.000 * | 0.000 * | 0.220 | 0.031 * | 0.106 | 0.144 |
| Treatment * Age | 0.166 | 0.029 | 0.000 * | 0.001 * | 0.075 | 0.240 | 0.007 * | 0.054 |
| Genotype * Treatment * Age | 0.701 | 0.000 * | 0.000 * | 0.757 | 0.000 * | 0.131 | 0.002 * | 0.066 |

## ONL cells measurements

| | Count (by position) | | | | | | | |
|---|---|---|---|---|---|---|---|---|
| | Inferior retina | | | | Superior retina | | | |
| | -4 | -3 | -2 | -1 | 1 | 2 | 3 | 4 |
| Genotype | 0.942 | 0.267 | 0.016 * | 0.338 | 0.495 | 0.428 | 0.101 | 0.837 |
| Treatment | 0.072 | 0.132 | 0.006 * | 0.264 | 0.383 | 0.004 * | 0.200 | 0.031 * |
| Age | 0.243 | 0.192 | 0.053 | 0.000 * | 0.053 | 0.108 | 0.100 | 0.090 |
| Genotype * Treatment | 0.830 | 0.133 | 0.590 | 0.206 | 0.357 | 0.455 | 0.045 * | 0.303 |
| Genotype * Age | 0.242 | 0.003 * | 0.000 * | 0.028 * | 0.028 * | 0.000 * | 0.000 * | 0.009 * |
| Treatment * Age | 0.005 * | 0.090 | 0.018 * | 0.171 | 0.003 * | 0.522 | 0.000 * | 0.011 * |
| Genotype * Treatment * Age | 0.919 | 0.480 | 0.016 * | 0.217 | 0.074 | 0.010 * | 0.452 | 0.042 * |

| | Stacking (by position) | | | | | | | | |
|---|---|---|---|---|---|---|---|---|---|
| | Inferior retina | | | | Superior retina | | | | Area |
| | -4 | -3 | -2 | -1 | 1 | 2 | 3 | 4 | |
| Genotype | 0.781 | 0.007 * | 0.289 | 0.000 * | 0.535 | 0.538 | 0.094 | 0.478 | 0.211 |
| Treatment | 0.536 | 0.003 * | 0.052 | 0.000 * | 0.034 * | 0.033 * | 0.265 | 0.137 | 0.000 * |
| Age | 0.061 | 0.140 | 0.005 * | 0.003 * | 0.004 * | 0.266 | 0.408 | 0.198 | 0.000 * |
| Genotype * Treatment | 0.275 | 0.020 * | 0.006 * | 0.255 | 0.002 * | 0.006 * | 0.010 * | 0.460 | 0.000 * |
| Genotype * Age | 0.044 | 0.004 * | 0.634 | 0.529 | 0.322 | 0.540 | 0.010 * | 0.279 | 0.002 * |
| Treatment * Age | 0.060 | 0.422 | 0.776 | 0.113 | 0.026 * | 0.759 | 0.333 | 0.174 | 0.002 * |
| Genotype * Treatment * Age | 0.029 * | 0.492 | 0.425 | 0.834 | 0.215 | 0.503 | 0.954 | 0.660 | 0.000 * |

## Visual Function

| | Retinal Function | | | | Spatial Frequency Thresholds | | | |
|---|---|---|---|---|---|---|---|---|
| | Amplitude | | Implicit time | | | | | |
| | B-wave | A-wave | B-wave | A-wave | Clockwise | Clock Counterwise | Up | Down |
| Genotype | 0.015 * | 0.000 * | 0.002 * | 0.000 * | 0.000 * | 0.000 * | 0.000 * | 0.000 * |
| Treatment | 0.000 * | 0.002 * | 0.000 * | 0.008 * | 0.000 * | 0.001 * | 0.000 * | 0.000 * |
| Age | 0.001 * | 0.000 * | 0.000 * | 0.000 * | 0.068 | 0.125 | 0.232 | 0.050 |
| Genotype * Treatment | 0.000 * | 0.000 * | 0.000 * | 0.000 * | 0.000 * | 0.000 * | 0.000 * | 0.000 * |
| Genotype * Age | 0.038 * | 0.003 * | 0.060 | 0.000 * | 0.270 | 0.117 | 0.532 | 0.348 |
| Treatment * Age | 0.062 | 0.220 | 0.021 * | 0.010 * | 0.483 | 0.719 | 0.114 | 0.906 |
| Genotype * Treatment * Age | 0.121 | 0.284 | 0.001 * | 0.039 * | 0.711 | 0.267 | 0.721 | 0.232 |
