# Supplementary material for: Human equivalent doses of l-DOPA rescues retinal morphology and visual function in a murine model of albinism
Source: Sci Rep. 2023 Oct 11;13:17173. doi: 10.1038/s41598-023-44373-3 (PMC10567794; doi:10.1038/s41598-023-44373-3)
Supplement: Supplementary file 9 — Supplementary Table 2. [file 41598_2023_44373_MOESM9_ESM.pdf]

WEEKS	L-DOPA (mg/kg)	RNFL								IPL							
		Pigmented				Albino				Pigmented				Albino			
		mean	SD	*n*	stats	mean	SD	*n*	stats	mean	SD	*n*	stats	mean	SD	*n*	stats
**4**	0	9.02	0.14	*4*		9.21	0.26	*16*		59.92	1.79	*5*		62.13	3.94	*18*	
	6.15	9.02	0.27	*14*	$ (vs week 16; 0.002)	9.01	0.50	*9*		58.34	3.47	*14*	$ (vs week 16; 0.000)	63.03	2.93	*9*	
	9.35	8.90	0.39	*12*		9.08	0.22	*6*		64.43	2.12	*13*		60.37	1.42	*6*	
	12.3	9.13	0.14	*8*		9.26	0.46	*13*		59.60	1.17	*8*		63.45	2.35	*13*	
**5**	0	9.27	0.42	*13*		9.25	0.46	*20*		60.83	2.52	*14*		62.50	3.74	*19*	
	6.15	9.23	0.34	*16*		9.09	0.39	*12*		59.04	2.35	*16*	$ (vs week 16; 0.022)	61.27	1.87	*12*	
	9.35	9.12	0.42	*24*		9.10	0.41	*11*		62.66	3.65	*24*		61.13	4.25	*11*	
	12.3	9.14	0.34	*6*		8.92	0.38	*23*		59.66	1.36	*8*		60.00	2.60	*23*	
**6**	0	9.30	0.38	*24*		9.00	0.49	*30*		62.03	2.68	*24*		60.48	2.75	*29*	
	6.15	9.23	0.38	*19*		8.93	0.37	*16*		59.99	2.28	*19*		60.43	1.75	*16*	
	9.35	9.27	0.31	*41*		9.12	0.46	*16*		62.06	2.85	*41*		60.74	4.38	*16*	
	13.5	9.24	0.29	*28*		9.05	0.33	*18*		60.45	1.86	*28*		61.12	2.77	*18*	
**12**	0	9.29	0.42	*25*		9.01	0.55	*20*		61.35	3.63	*25*		62.65	5.86	*20*	
	6.15	9.44	0.34	*16*		9.27	0.36	*10*		60.87	3.78	*16*		61.91	3.71	*10*	
	9.35	9.27	0.47	*25*		9.02	0.30	*13*		64.06	2.83	*25*		60.84	3.54	*13*	
	12.3	9.45	0.34	*25*		9.07	0.37	*17*		63.09	3.94	*25*		61.80	2.96	*17*	
**16**	0	9.57	0.27	*25*		9.07	0.60	*22*		60.95	3.28	*26*		60.72	3.19	*22*	
	6.15	9.65	0.27	*15*		9.14	0.32	*17*		63.17	3.13	*15*		60.86	3.30	*17*	
	9.35	9.35	0.45	*36*		9.34	0.27	*15*		62.72	2.86	*36*		64.33	3.33	*15*	
	12.3	9.42	0.34	*25*		9.07	0.48	*20*		62.85	3.08	*25*		61.19	2.21	*20*	

WEEKS	L-DOPA (mg/kg)	INL								OPL							
		Pigmented				Albino				Pigmented				Albino			
		mean	SD	*n*	stats	mean	SD	*n*	stats	mean	SD	*n*	stats	mean	SD	*n*	stats
**4**	0	27.23	1.38	*5*	$ (vs week 6; 0.010)<br>$ (vs week 12; 0.000)<br>$ (vs week 16; 0.003)	24.49	2.55	*19*		8.27	0.09	*5*	$ (vs week 12; 0.007)	8.10	0.35	*18*	
	6.15	26.43	1.90	*14*	$ (vs week 12; 0.000)<br>$ (vs week 16; 0.000)	28.56	1.67	*9*	# (0.016)<br>$ (vs week 6; 0.004)<br>$ (vs week 12; 0.000)<br>$ (vs week 16; 0.000)	8.00	0.45	*8*		7.94	0.42	*8*	
	9.35	25.82	0.99	*11*	$ (vs week 6; 0.034)<br>$ (vs week 12; 0.000)<br>$ (vs week 16; 0.002)	26.83	2.35	*6*	$ (vs week 5; 0.013)<br>$ (vs week 16; 0.007)	8.42	0.28	*11*	$ (vs week 6; 0.000)<br>$ (vs week 12; 0.000)<br>$ (vs week 16; 0.000)	8.03	0.23	*6*	
	12.3	27.39	1.73	*8*	$ (vs week 6; 0.001)<br>$ (vs week 12; 0.000)<br>$ (vs week 16; 0.000)	26.42	2.72	*13*		8.21	0.15	*8*	$ (vs week 12; 0.008)	8.05	0.25	*13*	
**5**	0	24.49	2.35	*14*	$ (vs week 12; 0.008)	22.65	1.90	*19*		8.09	0.29	*14*	$ (vs week 12; 0.002)	7.99	0.28	*20*	
	6.15	24.34	1.85	*16*	$ (vs week 16; 0.002)	24.66	1.64	*12*	$ (vs week 12; 0.001)	8.03	0.39	*16*	$ (vs week 16; 0.022)	7.97	0.33	*12*	
	9.35	24.39	1.56	*25*	$ (vs week 12; 0.001)	21.92	2.36	*11*		8.00	0.43	*24*		7.83	0.24	*11*	
	12.3	26.01	2.90	*8*	$ (vs week 12; 0.002)<br>$ (vs week 16; 0.022)	24.99	2.46	*23*		8.27	0.19	*8*	$ (vs week 12; 0.001)<br>$ (vs week 16; 0.034)	8.09	0.28	*22*	
**6**	0	22.28	1.81	*24*		24.20	2.58	*30*		7.74	0.41	*24*		8.02	0.32	*29*	
	6.15	23.46	2.20	*19*		23.98	1.42	*16*	$ (vs week 12; 0.004)	7.98	0.46	*19*		8.09	0.21	*16*	
	9.35	22.64	1.80	*40*		22.48	2.04	*16*		7.69	0.38	*40*		7.90	0.40	*15*	
	12.3	22.90	1.58	*28*		23.51	1.40	*18*		7.84	0.38	*28*		7.93	0.18	*13*	
**12**	0	21.34	1.62	*25*		23.27	3.55	*20*		7.52	0.45	*25*	# (0.013)	7.97	0.47	*20*	* (0.013)
	6.15	22.01	2.12	*16*		20.31	2.10	*10*		7.67	0.60	*16*		7.79	0.27	*8*	
	9.35	21.61	2.37	*25*		23.50	2.64	*13*		7.80	0.47	*25*		8.07	0.35	*13*	* (0.008)
	12.3	21.62	2.08	*25*		23.82	1.87	*17*		7.63	0.28	*26*		7.93	0.18	*17*	
**16**	0	22.07	1.94	*26*	# (0.007)	25.14	3.48	*22*	* (0.007)	7.78	0.45	*26*		7.95	0.41	*21*	
	6.15	20.86	1.62	*15*		23.42	1.76	*17*		7.49	0.37	*15*		7.94	0.19	*17*	
	9.35	22.27	2.31	*36*		22.05	2.63	*15*	# (0.044)	7.69	0.37	*36*		7.86	0.27	*15*	
	12.3	22.16	2.31	*25*		23.63	3.25	*20*		7.73	0.35	*25*		7.96	0.37	*20*	

Albino values — Pigmented Physiological values

WEEKS	L-DOPA (mg/kg)	ONL								IS							
		Pigmented				Albino				Pigmented				Albino			
**4**	0	61.30	5.24	*5*		65.10	2.70	*16*		7.63	0.22	*5*		8.89	1.03	*19*	$ (vs week 12; 0.002)<br>$ (vs week 16; 0.000)
	6.15	60.53	2.45	*14*		65.56	2.78	*9*	$ (vs week 12; 0.008)	7.16	0.28	*14*	$ (vs week 6; 0.000)<br>$ (vs week 12; 0.000)<br>$ (vs week 16; 0.000)	8.85	0.74	*9*	$ (vs week 12; 0.009)<br>$ (vs week 16; 0.019)
	9.35	63.27	1.02	*10*		63.06	1.86	*4*		7.88	0.53	*13*	$ (vs week 16; 0.027)	8.70	0.72	*6*	
	12.3	60.70	2.62	*7*		65.47	1.62	*13*	$ (vs week 6; 0.032)	7.14	0.21	*8*	$ (vs week 12; 0.000)<br>$ (vs week 16; 0.000)	9.12	0.83	*13*	$ (vs week 12; 0.001)
**5**	0	61.55	1.22	*11*		64.74	1.79	*18*		8.14	0.66	*14*		9.41	1.11	*20*	$ (vs week 16; 0.024)
	6.15	58.86	3.24	*16*		65.44	1.63	*10*	* (0.032)<br>$ (vs week 12; 0.005)	7.59	0.37	*16*	$ (vs week 16; 0.004)	9.76	0.70	*12*	* (0.007)
	9.35	61.95	1.43	*24*		60.93	2.13	*11*	# (0.006)	8.07	0.68	*24*	$ (vs week 16; 0.025)	9.65	1.05	*11*	
	12.3	58.20	1.21	*8*		62.95	1.80	*23*		7.31	0.30	*8*	$ (vs week 12; 0.004)<br>$ (vs week 16; 0.002)	9.49	1.01	*23*	* (0.007)<br>$ (vs week 12; 0.005)
**6**	0	60.68	1.39	*24*	# (0.000)	66.40	3.14	*28*	* (0.000)	8.18	0.69	*24*		9.14	1.01	*30*	$ (vs week 12; 0.009)<br>$ (vs week 16; 0.000)
	6.15	60.78	4.39	*19*		62.95	1.25	*14*		8.36	0.99	*19*		9.26	0.51	*16*	
	9.35	61.35	1.82	*41*		61.55	2.75	*15*	# (0.000)	8.34	0.77	*41*		9.71	1.03	*16*	* (0.001)
	13.5	60.04	2.12	*27*		61.80	2.76	*13*	# (0.000)	7.77	0.64	*28*	$ (vs week 12; 0.046)<br>$ (vs week 16; 0.019)	9.89	1.27	*17*	* (0.001)
**12**	0	60.46	2.38	*25*	# (0.000)	65.47	2.22	*19*	* (0.000)	8.66	0.88	*25*		10.50	1.20	*20*	* (0.000)
	6.15	59.82	4.03	*16*		60.99	1.67	*10*	# (0.000)	8.62	0.90	*16*		11.12	0.57	*10*	* (0.000)
	9.35	60.74	2.85	*25*		61.82	2.51	*7*		8.60	0.83	*25*		10.78	1.73	*13*	* (0.000)
	12.3	60.25	2.85	*25*		62.44	2.93	*11*		8.62	0.98	*26*		11.15	0.76	*17*	* (0.000)
**16**	0	60.91	2.95	*26*	# (0.000)	65.47	1.71	*18*	* (0.000)	8.61	0.66	*26*		10.81	1.02	*22*	* (0.000)
	6.15	61.63	3.06	*15*		62.70	1.72	*11*		8.79	0.58	*15*		10.93	1.61	*17*	* (0.000)
	9.35	62.37	1.47	*36*		63.46	1.92	*4*		8.97	0.71	*36*		10.27	1.57	*15*	* (0.005)
	12.3	60.29	2.13	*24*		62.62	1.86	*13*		8.57	0.57	*25*		10.60	1.35	*20*	* (0.000)
		mean	SD	*n*	stats	mean	SD	*n*	stats	mean	SD	*n*	stats	mean	SD	*n*	stats

WEEKS	L-DOPA (mg/kg)	OS								RPE							
		Pigmented				Albino				Pigmented				Albino			
**4**	0	28.57	0.25	*4*		26.55	0.92	*17*		19.94	1.61	*5*		22.59	1.55	*19*	
	6.15	27.00	1.32	*7*		28.65	0.95	*7*		19.38	0.80	*14*		22.88	1.75	*9*	
	9.35	31.00	0.65	*3*		27.56	0.34	*6*		20.74	1.35	*12*		21.50	0.73	*6*	
	12.3	27.02	1.50	*5*		27.57	0.97	*12*		20.04	1.21	*8*		22.24	1.42	*11*	
**5**	0	28.43	0.54	*8*	# (0.024)	26.13	1.16	*15*	* (0.024)	19.84	0.75	*13*	$ (vs week 16; 0.003)	22.38	1.33	*20*	* (0.002)<br>$ (vs week 12; 0.042)
	6.15	27.14	1.80	*11*		28.90	1.03	*11*	# (0.000)	19.42	1.28	*16*		22.35	1.80	*12*	* (0.025)
	9.35	29.64	1.38	*19*		26.25	0.92	*7*		20.43	1.30	*24*		21.57	1.72	*11*	
	12.3	27.24	1.03	*7*		27.38	1.25	*22*	# (0.000)	19.07	1.28	*8*		21.96	0.72	*19*	
**6**	0	28.29	1.41	*20*	# (0.000)	25.97	1.15	*21*	* (0.000)	20.27	1.27	*24*		23.05	1.84	*30*	* (0.000)
	6.15	27.02	1.05	*18*		28.73	1.17	*12*	# (0.000)	21.25	2.31	*19*		22.74	1.22	*16*	* (0.001)
	9.35	28.88	1.09	*38*		26.60	0.62	*13*		20.10	1.28	*41*		22.05	1.62	*15*	
	12.3	28.32	1.82	*26*		27.95	1.47	*14*	# (0.004)	19.93	1.31	*28*		22.23	1.55	*16*	
**12**	0	28.86	1.27	*23*	# (0.000)	26.33	1.05	*11*	* (0.000)	21.41	1.41	*25*		24.72	2.19	*20*	* (0.000)
	6.15	27.29	1.11	*16*		28.01	1.59	*10*		19.73	2.09	*16*	* (0.012)	23.22	1.46	*10*	
	9.35	28.22	1.32	*22*		28.00	1.74	*13*		20.99	1.24	*25*		23.67	1.43	*13*	* (0.046)
	12.3	27.65	1.29	*24*		28.82	1.24	*15*	# (0.001)	20.52	1.20	*25*		23.69	1.47	*9*	
**16**	0	29.06	0.95	*18*	# (0.000)	26.86	0.69	*11*	* (0.000)	21.98	1.72	*26*		23.57	1.50	*22*	
	6.15	27.11	1.00	*14*		28.44	1.54	*16*		20.82	1.26	*15*		24.24	1.57	*17*	* (0.002)
	9.35	28.73	1.09	*33*		27.66	1.81	*12*		21.15	1.48	*35*		23.70	1.49	*13*	
	12.3	27.39	1.25	*21*		28.15	1.30	*14*		20.18	1.16	*25*		23.62	0.99	*16*	
		mean	SD	*n*	stats	mean	SD	*n*	stats	mean	SD	*n*	stats	mean	SD	*n*	stats

Albino values	Pigmented<br>Physiological values
