# Supplementary material for: Human equivalent doses of l-DOPA rescues retinal morphology and visual function in a murine model of albinism
Source: Sci Rep. 2023 Oct 11;13:17173. doi: 10.1038/s41598-023-44373-3 (PMC10567794; doi:10.1038/s41598-023-44373-3)
Supplement: Supplementary file 10 — Supplementary Table 3. [file 41598_2023_44373_MOESM10_ESM.pdf]

WEEKS	L-DOPA (mg/kg)	Cell Area									
		Pigmented					Albino				
**7**	0	12.36	3.11	*200*	*4*	# (0.000)<br>$ (vs week 11; 0.000)	15.24	6.26	*500*	*10*	* (0.000)<br>$ (vs week 15; 0.000)
	6.15	12.63	3.31	*200*	*4*		11.72	3.11	*200*	*4*	# (0.000)
	9.35	12.10	2.76	*300*	*6*		11.53	4.28	*200*	*4*	# (0.000)
	13.5	11.73	3.58	*200*	*4*		11.61	2.73	*200*	*4*	# (0.000)<br>$ (vs week 11; 0.033)
**11**	0	10.85	3.50	*150*	*3*	# (0.000)	14.55	5.09	*400*	*8*	# (0.000)<br>$ (vs week 15; 0.000)
	6.15	11.44	3.11	*200*	*4*		12.06	3.59	*200*	*4*	# (0.000)
	9.35	11.43	3.37	*200*	*4*		11.76	3.94	*200*	*4*	# (0.000)
	12.3	11.97	3.65	*200*	*4*	* (0.031)	10.44	2.74	*200*	*4*	# (0.000)
**15**	0	11.72	3.83	*600*	*12*	# (0.000)	13.62	6.15	*300*	*6*	* (0.000)
	6.15	12.00	3.42	*200*	*4*		11.32	3.94	*400*	*4*	# (0.000)
	9.35	12.53	3.70	*300*	*6*		11.66	3.72	*200*	*4*	# (0.001)
	12.3	11.34	3.44	*200*	*4*		11.23	3.12	*200*	*4*	# (0.000)
		mean	SD	*cell count*	*n*	stats	mean	SD	*cell count*	*n*	stats

Albino values — Pigmented Physiological values
