# Supplementary material for: Human equivalent doses of l-DOPA rescues retinal morphology and visual function in a murine model of albinism
Source: Sci Rep. 2023 Oct 11;13:17173. doi: 10.1038/s41598-023-44373-3 (PMC10567794; doi:10.1038/s41598-023-44373-3)
Supplement: Supplementary file 11 — Supplementary Table 4. [file 41598_2023_44373_MOESM11_ESM.pdf]

INFERIOR RETINA

WEEKS	L-DOPA (mg/kg)	Position -4							
		Pigmented				Albino			
**7**	0	136.67	12.66	*3*		130.33	21.94	*3*	
	6.15	202.67	10.50	*3*		180.33	18.90	*3*	
	9.35	192.00	80.89	*3*		183.00	4.36	*3*	
	13.5	163.33	22.19	*3*		172.00	21.66	*3*	
**11**	0	181.00	57.11	*3*		180.00	27.22	*3*	
	6.15	194.33	67.86	*3*		174.33	8.33	*3*	
	9.35	144.67	58.59	*3*		153.67	20.31	*3*	
	12.3	250.33	3.79	*3*		216.67	5.51	*3*	
**15**	0	168.33	42.36	*3*		202.67	23.35	*3*	
	6.15	164.33	47.72	*3*		170.33	6.66	*3*	
	9.35	162.00	10.82	*3*		169.33	25.42	*3*	
	12.3	164.00	55.00	*3*		198.33	2.31	*3*	
		mean	SD	*n*	stats	mean	SD	*n*	stats

WEEKS	L-DOPA (mg/kg)	Position -3							
		Pigmented				Albino			
**7**	0	282.33	19.04	*3*		260.00	7.00	*3*	
	6.15	335.33	25.72	*3*		248.67	14.36	*3*	
	9.35	328.67	92.82	*3*		243.00	9.54	*3*	
	13.5	350.67	44.79	*3*		282.33	47.82	*3*	
**11**	0	298.67	32.59	*3*		296.33	14.57	*3*	
	6.15	330.00	34.70	*3*		325.67	17.79	*3*	
	9.35	268.00	51.16	*3*		292.33	24.01	*3*	
	12.3	347.00	22.87	*3*		362.00	7.21	*3*	
**15**	0	292.00	31.19	*3*		374.33	85.65	*3*	
	6.15	330.00	104.01	*3*		268.67	52.05	*3*	
	9.35	256.00	57.94	*3*		312.67	16.62	*3*	
	12.3	280.33	36.30	*3*		291.33	10.97	*3*	
		mean	SD	*n*	stats	mean	SD	*n*	stats

WEEKS	L-DOPA (mg/kg)	Position -2							
		Pigmented				Albino			
**7**	0	383.67	22.50	*3*		313.67	48.95	*3*	
	6.15	334.67	17.21	*3*		302.67	66.20	*3*	
	9.35	347.00	33.87	*3*		278.33	14.50	*3*	
	13.5	392.00	13.00	*3*	$ (vs week 15; 0.026)	293.00	31.05	*3*	
**11**	0	346.67	35.36	*3*		311.33	23.01	*3*	
	6.15	334.67	36.02	*3*		316.33	7.09	*3*	
	9.35	302.33	27.50	*3*		283.00	36.10	*3*	
	12.3	368.33	14.01	*3*		384.33	12.01	*3*	
**15**	0	306.00	27.62	*3*		372.33	50.36	*3*	
	6.15	332.33	52.69	*3*		269.67	20.74	*3*	
	9.35	278.33	40.77	*3*		336.67	16.65	*3*	
	12.3	277.00	45.31	*3*		308.67	11.37	*3*	
		mean	SD	*n*	stats	mean	SD	*n*	stats

WEEKS	L-DOPA (mg/kg)	Position -1							
		Pigmented				Albino			
**7**	0	228.00	54.74	*3*		236.33	25.03	*3*	
	6.15	278.00	36.59	*3*		200.33	50.40	*3*	
	9.35	219.67	129.39	*3*		259.00	18.33	*3*	
	13.5	256.00	37.03	*3*		230.00	28.16	*3*	
**11**	0	310.00	6.24	*3*		283.67	12.90	*3*	
	6.15	257.33	56.05	*3*		278.67	43.10	*3*	
	9.35	242.00	37.99	*3*		239.00	48.38	*3*	
	12.3	297.67	4.51	*3*		285.67	10.60	*3*	
**15**	0	206.33	6.66	*3*		233.67	18.88	*3*	
	6.15	195.67	9.02	*3*		188.67	48.42	*3*	
	9.35	207.33	31.56	*3*		279.00	10.82	*3*	
	12.3	195.33	21.50	*3*		291.67	13.01	*3*	
		mean	SD	*n*	stats	mean	SD	*n*	stats

PERIPHERAL RETINA

CENTRAL RETINA

SUPERIOR RETINA

WEEKS	L-DOPA (mg/kg)	Position 4							
		Pigmented				Albino			
**7**	0	174.67	9.61	*3*		130.33	7.64	*3*	
	6.15	177.33	18.82	*3*		161.33	38.66	*3*	
	9.35	184.67	23.07	*3*		155.33	12.01	*3*	
	13.5	140.67	18.15	*3*		177.00	11.53	*3*	
**11**	0	177.67	53.46	*3*		137.67	32.81	*3*	
	6.15	168.67	37.75	*3*		164.00	10.58	*3*	
	9.35	160.00	66.78	*3*		138.33	18.77	*3*	
	12.3	233.67	9.29	*3*		241.00	14.42	*3*	
**15**	0	117.33	29.19	*3*		181.67	13.05	*3*	
	6.15	162.67	25.15	*3*		159.33	13.43	*3*	
	9.35	125.00	30.35	*3*		164.67	13.20	*3*	
	12.3	144.33	42.10	*3*		165.00	8.89	*3*	
		mean	SD	*n*	stats	mean	SD	*n*	stats

WEEKS	L-DOPA (mg/kg)	Position 3							
		Pigmented				Albino			
**7**	0	294.67	26.31	*3*		216.67	43.15	*3*	$ (vs week 15; 0.001)
	6.15	323.33	8.08	*3*		253.00	15.00	*3*	
	9.35	320.33	53.58	*3*		256.67	9.29	*3*	
	13.5	361.00	43.71	*3*	$ (vs week 15; 0.011)	299.00	26.96	*3*	
**11**	0	312.33	19.60	*3*		269.00	24.76	*3*	
	6.15	341.67	60.62	*3*		278.33	19.04	*3*	
	9.35	292.00	39.23	*3*		310.67	46.72	*3*	
	12.3	338.67	11.85	*3*		363.00	7.81	*3*	
**15**	0	299.33	61.45	*3*		368.67	19.30	*3*	
	6.15	325.33	56.05	*3*		300.33	47.25	*3*	
	9.35	265.33	48.79	*3*		314.67	6.43	*3*	
	12.3	229.00	34.39	*3*		304.00	7.21	*3*	
		mean	SD	*n*	stats	mean	SD	*n*	stats

WEEKS	L-DOPA (mg/kg)	Position 2							
		Pigmented				Albino			
**7**	0	320.00	23.81	*3*		257.00	10.00	*3*	
	6.15	345.67	22.48	*3*		356.00	28.93	*3*	
	9.35	343.00	22.91	*3*		258.67	14.57	*3*	
	13.5	346.67	27.97	*3*		300.00	21.66	*3*	
**11**	0	331.33	27.47	*3*		317.67	16.26	*3*	
	6.15	387.00	19.47	*3*		308.00	10.58	*3*	
	9.35	320.00	44.40	*3*		304.33	44.66	*3*	
	12.3	347.67	25.11	*3*		383.33	12.22	*3*	
**15**	0	278.67	55.43	*3*		360.00	10.44	*3*	
	6.15	340.00	36.59	*3*		323.00	30.20	*3*	
	9.35	269.33	99.49	*3*		349.67	26.58	*3*	
	12.3	315.00	54.37	*3*		347.00	23.90	*3*	
		mean	SD	*n*	stats	mean	SD	*n*	stats

WEEKS	L-DOPA (mg/kg)	Position 1							
		Pigmented				Albino			
**7**	0	261.00	7.00	*3*		306.00	40.60	*3*	
	6.15	260.00	24.88	*3*		169.67	13.20	*3*	# (0.013)
	9.35	303.67	46.72	*3*		269.00	7.94	*3*	
	13.5	244.33	13.87	*3*		210.33	69.76	*3*	
**11**	0	259.67	35.84	*3*		264.67	45.21	*3*	
	6.15	261.67	52.84	*3*		273.33	12.50	*3*	
	9.35	206.67	86.22	*3*		227.00	46.87	*3*	
	12.3	248.00	4.58	*3*		299.00	2.65	*3*	
**15**	0	239.67	51.73	*3*		221.67	6.66	*3*	
	6.15	223.00	10.15	*3*		238.00	33.05	*3*	
	9.35	197.33	20.79	*3*		268.67	50.82	*3*	
	12.3	213.33	26.35	*3*		243.67	13.43	*3*	
		mean	SD	*n*	stats	mean	SD	*n*	stats

Albino values — Pigmented Physiological values
