# Supplementary material for: Human equivalent doses of l-DOPA rescues retinal morphology and visual function in a murine model of albinism
Source: Sci Rep. 2023 Oct 11;13:17173. doi: 10.1038/s41598-023-44373-3 (PMC10567794; doi:10.1038/s41598-023-44373-3)
Supplement: Supplementary file 12 — Supplementary Table 5. [file 41598_2023_44373_MOESM12_ESM.pdf]

INFERIOR RETINA

WEEKS	L-DOPA (mg/kg)	Position -4 Pigmented				Albino			
		mean	SD	*n*	stats	mean	SD	*n*	stats
**7**	0	5.33	1.53	*3*		6.33	2.31	*3*	
	6.15	7.00	0.00	*3*		6.00	0.00	*3*	
	9.35	7.33	2.08	*3*		7.67	0.58	*3*	
	13.5	5.33	0.58	*3*		8.33	0.58	*3*	
**11**	0	6.67	0.58	*3*		5.67	0.58	*3*	
	6.15	6.00	1.00	*3*		6.33	0.58	*3*	
	9.35	6.00	1.00	*3*		5.67	0.58	*3*	
	12.3	8.00	2.00	*3*		5.33	0.58	*3*	
**15**	0	5.67	1.15	*3*		6.67	0.58	*3*	
	6.15	7.00	0.00	*3*		6.00	1.00	*3*	
	9.35	6.00	0.00	*3*		5.33	0.58	*3*	
	12.3	5.00	1.00	*3*		5.67	0.58	*3*	

WEEKS	L-DOPA (mg/kg)	Position -3 Pigmented				Albino			
		mean	SD	*n*	stats	mean	SD	*n*	stats
**7**	0	10.33	0.58	*3*		10.00	2.00	*3*	
	6.15	11.00	1.00	*3*		7.67	0.58	*3*	
	9.35	11.33	0.58	*3*		10.00	1.00	*3*	
	13.5	11.67	0.58	*3*		9.00	0.00	*3*	
**11**	0	11.00	0.00	*3*		11.67	0.58	*3*	
	6.15	9.67	1.15	*3*		9.67	1.15	*3*	
	9.35	10.33	0.58	*3*		9.33	1.53	*3*	
	12.3	10.67	1.15	*3*		11.00	1.00	*3*	
**15**	0	9.67	0.58	*3*		11.33	0.58	*3*	
	6.15	9.33	0.58	*3*		8.67	1.15	*3*	
	9.35	10.00	1.00	*3*		11.33	3.21	*3*	
	12.3	10.33	2.31	*3*		9.33	0.58	*3*	

WEEKS	L-DOPA (mg/kg)	Position -2 Pigmented				Albino			
		mean	SD	*n*	stats	mean	SD	*n*	stats
**7**	0	10.67	1.53	*3*		11.67	0.58	*3*	
	6.15	11.00	1.00	*3*		11.33	0.58	*3*	
	9.35	10.67	0.58	*3*		10.33	0.58	*3*	
	13.5	11.33	2.08	*3*		10.67	0.58	*3*	
**11**	0	10.67	0.58	*3*		13.33	0.58	*3*	
	6.15	10.67	2.31	*3*		11.00	0.00	*3*	
	9.35	10.33	0.58	*3*		10.33	1.53	*3*	
	12.3	10.33	0.58	*3*		10.33	1.53	*3*	
**15**	0	9.33	0.58	*3*		12.00	1.00	*3*	
	6.15	10.33	0.58	*3*		8.67	1.53	*3*	
	9.35	9.00	2.00	*3*		10.33	2.52	*3*	
	12.3	10.33	1.15	*3*		8.33	0.58	*3*	

WEEKS	L-DOPA (mg/kg)	Position -1 Pigmented				Albino			
		mean	SD	*n*	stats	mean	SD	*n*	stats
**7**	0	9.33	1.53	*3*		12.00	0.00	*3*	
	6.15	7.67	2.08	*3*		8.33	1.53	*3*	
	9.35	8.33	1.53	*3*		9.67	0.58	*3*	
	13.5	8.67	0.58	*3*		9.00	1.00	*3*	
**11**	0	9.67	0.58	*3*		10.67	0.58	*3*	
	6.15	7.00	0.00	*3*		8.33	1.53	*3*	
	9.35	7.67	2.52	*3*		8.33	1.53	*3*	
	12.3	9.33	1.53	*3*		9.00	1.73	*3*	
**15**	0	7.67	1.15	*3*		9.67	0.58	*3*	
	6.15	8.00	1.00	*3*		8.67	1.53	*3*	
	9.35	6.00	1.00	*3*		8.33	0.58	*3*	
	12.3	6.67	2.08	*3*		7.67	0.58	*3*	

PERIPHERAL RETINA

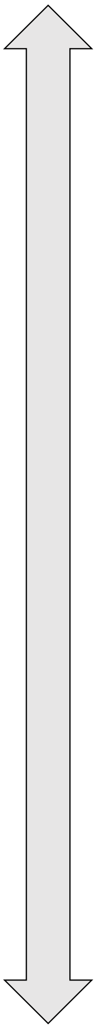

CENTRAL RETINA

SUPERIOR RETINA

WEEKS	L-DOPA (mg/kg)	Position 4 Pigmented				Albino			
		mean	SD	*n*	stats	mean	SD	*n*	stats
**7**	0	6.67	0.58	*3*		7.00	2.00	*3*	
	6.15	5.67	1.15	*3*		5.67	1.53	*3*	
	9.35	7.00	0.00	*3*		6.67	0.58	*3*	
	13.5	5.67	0.58	*3*		7.33	0.58	*3*	
**11**	0	6.00	1.00	*3*		6.00	1.00	*3*	
	6.15	6.00	1.00	*3*		5.67	0.58	*3*	
	9.35	5.33	1.53	*3*		5.00	1.00	*3*	
	12.3	7.00	2.65	*3*		6.00	0.00	*3*	
**15**	0	6.67	1.53	*3*		7.00	1.00	*3*	
	6.15	5.67	1.15	*3*		5.33	0.58	*3*	
	9.35	7.33	0.58	*3*		5.33	0.58	*3*	
	12.3	5.67	0.58	*3*		5.33	0.58	*3*	

WEEKS	L-DOPA (mg/kg)	Position 3 Pigmented				Albino			
		mean	SD	*n*	stats	mean	SD	*n*	stats
**7**	0	9.33	0.58	*3*		9.33	1.53	*3*	
	6.15	10.33	1.53	*3*		7.33	2.08	*3*	
	9.35	9.00	0.00	*3*		9.00	1.00	*3*	
	13.5	10.33	0.58	*3*		10.00	1.00	*3*	
**11**	0	9.33	0.58	*3*		11.00	1.00	*3*	
	6.15	10.00	1.73	*3*		8.67	0.58	*3*	
	9.35	9.00	1.73	*3*		10.67	1.53	*3*	
	12.3	8.67	0.58	*3*		10.00	1.00	*3*	
**15**	0	9.67	0.58	*3*		12.00	0.00	*3*	
	6.15	9.67	2.52	*3*		10.00	2.00	*3*	
	9.35	8.33	1.53	*3*		10.67	0.58	*3*	
	12.3	9.00	1.00	*3*		9.67	0.58	*3*	

WEEKS	L-DOPA (mg/kg)	Position 2 Pigmented				Albino			
		mean	SD	*n*	stats	mean	SD	*n*	stats
**7**	0	10.67	0.58	*3*		11.33	0.58	*3*	
	6.15	11.00	0.00	*3*		10.33	1.53	*3*	
	9.35	10.33	0.58	*3*		9.67	0.58	*3*	
	13.5	11.33	1.53	*3*		9.33	0.58	*3*	
**11**	0	10.33	0.58	*3*		11.33	1.15	*3*	
	6.15	11.67	0.58	*3*		10.00	1.00	*3*	
	9.35	9.67	2.31	*3*		11.00	1.00	*3*	
	12.3	10.67	0.58	*3*		11.00	1.00	*3*	
**15**	0	10.33	0.58	*3*		11.67	0.58	*3*	
	6.15	11.00	0.00	*3*		9.67	2.08	*3*	
	9.35	9.33	1.53	*3*		9.67	0.58	*3*	
	12.3	11.00	1.00	*3*		9.67	0.58	*3*	

WEEKS	L-DOPA (mg/kg)	Position 1 Pigmented				Albino			
		mean	SD	*n*	stats	mean	SD	*n*	stats
**7**	0	8.67	0.58	*3*		12.33	0.58	*3*	
	6.15	9.33	0.58	*3*		6.67	0.58	*3*	# (0.008)
	9.35	9.00	1.73	*3*		10.33	1.15	*3*	
	13.5	8.67	0.58	*3*		7.33	0.58	*3*	
**11**	0	9.67	1.53	*3*		9.33	0.58	*3*	
	6.15	9.33	1.53	*3*		8.33	1.53	*3*	
	9.35	8.33	2.08	*3*		8.67	1.15	*3*	
	12.3	10.33	2.08	*3*		10.00	1.73	*3*	
**15**	0	7.33	1.53	*3*		10.33	0.58	*3*	
	6.15	8.33	0.58	*3*		8.00	1.73	*3*	
	9.35	7.67	0.58	*3*		8.00	1.00	*3*	
	12.3	7.00	2.65	*3*		7.33	0.58	*3*	

Albino values — Pigmented Physiological values
