# Supplementary material for: Human equivalent doses of l-DOPA rescues retinal morphology and visual function in a murine model of albinism
Source: Sci Rep. 2023 Oct 11;13:17173. doi: 10.1038/s41598-023-44373-3 (PMC10567794; doi:10.1038/s41598-023-44373-3)
Supplement: Supplementary file 13 — Supplementary Table 6. [file 41598_2023_44373_MOESM13_ESM.pdf]

WEEKS	L-DOPA (mg/kg)	Amplitude of b-wave (µV)								Amplitude of a-wave (µV)							
		Pigmented				Albino				Pigmented				Albino			
**4**	0	303.63	37.83	6		164.14	31.24	8		-178.62	14.33	5	# (0.000)	-95.62	29.01	16	* (0.000)
	6.15	330.26	95.48	7		341.44	21.47	7	# (0.000)	-161.12	39.76	6		-198.33	25.52	7	# (0.000)<br>$ (vs week 16; 0.003)
	9.35	285.90	86.37	14		347.71	65.79	7	# (0.009)	-137.38	18.04	12		-196.25	53.96	8	# (0.000)<br>$ (vs week 16; 0.012)
	12.3	273.59	80.38	9		323.61	68.57	24	# (0.000)	-132.61	29.67	9		-179.12	48.85	24	# (0.000)<br>$ (vs week 12; 0.017)
**5**	0	297.03	66.26	16	# (0.012)	173.01	39.77	12	* (0.012)	-158.21	30.19	12	# (0.001)	-105.67	19.34	22	* (0.001)
	6.15	244.87	68.82	15		248.30	54.03	11		-150.91	31.03	9		-174.58	54.16	18	# (0.000)
	9.35	270.28	70.41	31		245.69	74.01	13		-158.47	44.73	20		-137.45	37.49	13	
	12.3	299.26	88.21	22		288.20	48.64	23	# (0.001)	-163.18	42.72	16		-162.41	35.56	20	# (0.000)
**6**	0	309.92	79.74	28	# (0.000)	149.04	33.94	22	* (0.000)	-160.65	25.43	22	# (0.000)	-81.68	29.44	19	* (0.000)
	6.15	255.29	81.05	20		313.87	79.40	21	# (0.000)	-151.72	33.90	12		-159.45	44.77	22	# (0.000)
	9.35	280.26	79.50	42		318.56	69.78	20	# (0.000)	-142.40	28.81	35		-162.60	37.19	20	# (0.000)
	13.5	298.47	72.70	31		298.51	59.66	27	# (0.000)	-146.66	32.93	26		-160.94	31.38	24	# (0.000)
**12**	0	259.72	66.86	25	# (0.009)	148.07	28.73	9	* (0.009)	-146.03	29.90	12	# (0.003)	-89.63	26.54	12	* (0.003)
	6.15	268.26	70.22	16		323.13	90.52	10	# (0.000)	-147.20	35.08	10		-141.56	34.55	10	
	9.35	232.49	54.08	36		317.55	81.79	18	# (0.000)	-127.23	18.07	24		-162.74	19.66	16	# (0.000)
	12.3	248.86	64.01	28		290.57	74.02	26	# (0.000)	-147.52	28.93	16		-138.18	34.85	26	# (0.006)
**16**	0	276.52	70.01	21	# (0.001)	163.52	38.77	14	* (0.001)	-152.80	29.58	10	# (0.001)	-94.10	12.57	13	* (0.001)
	6.15	254.21	41.15	16		282.82	67.83	19	# (0.000)	-119.84	12.56	13		-129.85	29.17	19	
	9.35	270.37	80.80	34		263.73	64.50	22	# (0.005)	-142.38	22.39	27		-131.20	30.46	22	
	12.3	267.37	64.33	27		301.38	74.29	26	# (0.000)	-153.50	36.42	26		-153.73	41.14	24	# (0.000)
		mean	SD	*n*	stats	mean	SD	*n*	stats	mean	SD	*n*	stats	mean	SD	*n*	stats

WEEKS	L-DOPA (mg/kg)	Implicit time for b-wave (ms)								Implicit time for a-wave (ms)							
		Pigmented				Albino				Pigmented				Albino			
**4**	0	46.40	2.21	4		38.62	5.21	20		15.07	1.32	6	# (0.000)	10.49	1.91	20	* (0.000)
	6.15	39.04	2.59	9		40.35	3.89	8		13.57	0.63	9		11.59	0.79	8	
	9.35	42.00	3.60	13		44.45	2.12	8		13.91	0.62	14		13.75	1.15	8	# (0.003)
	12.3	46.29	8.15	9		44.88	3.28	24	* (0.044)<br>$ (vs week 12; 0.000)<br>$ (vs week 16; 0.016)	14.73	1.34	9		13.51	1.00	24	# (0.000)<br>$ (vs week 6; 0.001)<br>$ (vs week 12; 0.010)
**5**	0	44.71	3.85	16		41.00	5.31	31	$ (vs week 12; 0.000)	14.63	1.45	15	# (0.003)	12.18	1.78	31	* (0.003)<br>$ (vs week 12; 0.035)
	6.15	39.44	2.90	10		42.80	7.48	18		13.91	0.83	11		13.66	1.55	18	$ (vs week 12; 0.001)
	9.35	43.21	5.45	28		42.82	4.66	12		14.22	1.85	28		12.90	1.24	12	
	12.3	40.37	5.82	22		41.16	5.95	24		12.94	1.75	21		12.67	1.57	24	
**6**	0	41.10	4.52	27		39.96	5.39	40	$ (vs week 12; 0.003)	13.68	1.96	24	# (0.000)	11.46	1.64	39	* (0.000)
	6.15	39.58	3.18	19		38.92	4.69	22		13.38	0.75	15		12.06	1.60	22	
	9.35	41.01	4.33	37		40.02	4.18	20		14.21	1.97	39		11.87	1.72	20	
	12.3	40.67	5.65	30		39.63	4.92	27		12.97	1.97	28		11.17	2.20	27	* (0.000)
**12**	0	42.13	5.03	24	# (0.000)	34.47	3.79	24	* (0.000)	13.90	1.85	23	# (0.000)	10.32	1.49	24	* (0.000)
	6.15	39.15	4.08	11		36.82	3.05	10		13.67	1.80	12		10.47	1.54	10	* (0.000)
	9.35	41.92	4.36	35		38.18	3.79	18		13.94	1.81	35		11.42	1.61	18	* (0.002)
	12.3	40.92	4.96	28		37.22	4.72	26		13.36	1.87	25		11.43	1.05	26	* (0.000)
**16**	0	41.08	5.03	24		37.95	5.00	26		13.70	1.59	26	# (0.004)	11.50	1.49	26	* (0.004)
	6.15	36.08	1.97	15		39.27	4.46	20		12.39	0.51	10		11.50	1.89	20	* (0.015)
	9.35	40.35	5.15	35		38.75	4.82	22		13.67	2.02	34		12.03	1.50	22	
	12.3	41.47	5.62	27		38.58	5.00	26		13.57	2.16	27		11.62	1.46	26	* (0.011)
		mean	SD	*n*	stats	mean	SD	*n*	stats	mean	SD	*n*	stats	mean	SD	*n*	stats

Albino values — Pigmented Physiological values

* Statistical differences with untreated WT

Statistical differences with untreated OCA1

$ Statistical differences across time
