# Supplementary material for: Human equivalent doses of l-DOPA rescues retinal morphology and visual function in a murine model of albinism
Source: Sci Rep. 2023 Oct 11;13:17173. doi: 10.1038/s41598-023-44373-3 (PMC10567794; doi:10.1038/s41598-023-44373-3)
Supplement: Supplementary file 14 — Supplementary Table 7. [file 41598_2023_44373_MOESM14_ESM.pdf]

WEEKS	L-DOPA (mg/kg)	Clockwise								Counter-clockwise							
		Pigmented				Albino				Pigmented				Albino			
**7**	0	0.44	0.07	*11*	# (0.000)	0.15	0.08	*14*	* (0.000)	0.49	0.09	*10*	# (0.000)	0.13	0.07	*14*	* (0.000)
	6.15	0.42	0.03	*7*		0.31	0.09	*8*	* (0.007)	0.40	0.05	*7*		0.27	0.11	*12*	* (0.020)
	9.35	0.43	0.06	*10*		0.31	0.06	*7*	* (0.000)	0.41	0.03	*11*		0.26	0.12	*11*	* (0.000)
	13.5	0.46	0.09	*4*		0.28	0.04	*6*	* (0.010)	0.53	0.18	*8*		0.26	0.11	*6*	* (0.021)
**11**	0	0.44	0.06	*22*	# (0.000)	0.19	0.07	*9*	* (0.000)	0.46	0.09	*23*	# (0.000)	0.16	0.08	*9*	* (0.000)
	6.15	0.38	0.03	*6*		0.33	0.08	*6*	* (0.037)	0.42	0.10	*5*		0.28	0.10	*7*	
	9.35	0.46	0.08	*20*		0.37	0.10	*11*		0.42	0.07	*20*		0.32	0.12	*10*	# (0.033)
	12.3	0.51	0.09	*10*		0.40	0.09	*8*	# (0.009)	0.49	0.07	*10*		0.34	0.09	*9*	# (0.007)
**15**	0	0.43	0.09	*17*	# (0.000)	0.17	0.07	*9*	* (0.000)	0.46	0.08	*16*	# (0.000)	0.17	0.06	*9*	* (0.000)
	6.15	0.40	0.03	*5*		0.35	0.09	*4*		0.43	0.09	*4*		0.30	0.11	*5*	
	9.35	0.47	0.08	*14*		0.34	0.03	*5*		0.46	0.08	*14*		0.36	0.05	*5*	
	12.3	0.44	0.06	*14*		0.38	0.14	*6*		0.43	0.06	*14*		0.38	0.16	*9*	
		mean	SD	*n*	stats	mean	SD	*n*	stats	mean	SD	*n*	stats	mean	SD	*n*	stats

WEEKS	L-DOPA (mg/kg)	Up								Down							
		Pigmented				Albino				Pigmented				Albino			
**7**	0	0.44	0.10	*10*	# (0.000)	0.18	0.07	*14*	* (0.000)	0.50	0.10	*11*	# (0.000)	0.25	0.08	*14*	* (0.000)
	6.15	0.37	0.07	*6*		0.28	0.10	*12*		0.43	0.06	*5*		0.43	0.14	*12*	
	9.35	0.45	0.09	*13*		0.33	0.03	*12*	# (0.017)	0.48	0.08	*13*		0.43	0.10	*12*	# (0.003)
	12.3	0.47	0.06	*6*		0.36	0.09	*7*	# (0.020)	0.56	0.09	*6*		0.35	0.09	*6*	# (0.005)
**11**	0	0.45	0.09	*22*	# (0.000)	0.21	0.07	*9*	* (0.000)	0.49	0.10	*21*	# (0.000)	0.26	0.08	*9*	* (0.000)
	6.15	0.43	0.18	*4*		0.33	0.12	*7*		0.45	0.05	*6*		0.32	0.12	*7*	* (0.043)
	9.35	0.42	0.08	*17*		0.33	0.07	*12*		0.46	0.10	*17*		0.41	0.14	*11*	
	12.3	0.49	0.13	*12*		0.38	0.05	*9*	# (0.036)	0.47	0.09	*13*		0.41	0.13	*9*	
**15**	0	0.43	0.14	*17*	# (0.000)	0.22	0.06	*9*	* (0.000)	0.43	0.11	*15*		0.26	0.04	*8*	
	6.15	0.52	0.07	*5*		0.35	0.08	*5*		0.40	0.03	*6*		0.32	0.05	*4*	
	9.35	0.39	0.07	*12*		0.37	0.08	*5*		0.39	0.11	*13*		0.40	0.18	*5*	
	12.3	0.42	0.08	*14*		0.37	0.11	*13*		0.47	0.07	*13*		0.41	0.11	*13*	
		mean	SD	*n*	stats	mean	SD	*n*	stats	mean	SD	*n*	stats	mean	SD	*n*	stats

Albino values — Pigmented Physiological values
