# Supplementary material for: Human equivalent doses of l-DOPA rescues retinal morphology and visual function in a murine model of albinism
Source: Sci Rep. 2023 Oct 11;13:17173. doi: 10.1038/s41598-023-44373-3 (PMC10567794; doi:10.1038/s41598-023-44373-3)
Supplement: Supplementary file 15 — Supplementary Table 8. [file 41598_2023_44373_MOESM15_ESM.pdf]

	L-DOPA (mg/kg)	PEDF							
		Week 6				Week 16			
Pigmented	0	1.00	0	*7*		1.06	0.30	*17*	
Albino	0	0.14	0.07	*4*	*	0.12	0.09	*7*	*
	6.15	0.44	0.44	*3*		0.39	0.48	*6*	*
	9.35	0.98	0.16	*4*	#	0.41	0.55	*7*	*
	12.3	1.31	0.86	*3*	#	0.15	0.14	*7*	*
		mean	SD	*n*	stats	mean	SD	*n*	stats

	L-DOPA (mg/kg)	Syntaxin 3							
		Week 6				Week 16			
Pigmented	0	1.00	0	*7*		1.00	0.34	*8*	
Albino	0	0.36	0.22	*5*	*	0.35	0.20	*4*	*
	6.15	0.60	0.44	*3*		0.21	0.09	*2*	*
	9.35	0.48	0.14	*3*		0.19	0.11	*3*	*
	12.3	0.60	0.31	*3*		0.32	0.13	*4*	*
		mean	SD	*n*	stats	0.13	SD	*n*	stats

	L-DOPA (mg/kg)	class-III β-tubulin							
		Week 6				Week 16			
Pigmented	0	1.00	0	*7*		1.00	0.19	*8*	
Albino	0	0.31	0.25	*4*	*	0.11	0.09	*8*	*
	6.15	0.62	0.30	*3*		0.96	1.28	*4*	
	9.35	0.48	0.25	*3*		0.55	0.59	*4*	
	12.3	0.54	0.27	*3*		0.80	0.65	*3*	
		mean	SD	*n*	stats	mean	SD	*n*	stats

* Statistical differences with untreated WT

Statistical differences with untreated OCA1
